# A Senomorphlytic Three‐Drug Combination Discovered in *Salsola collina* for Delaying Aging Phenotypes and Extending Healthspan

**DOI:** 10.1002/advs.202401862

**Published:** 2024-07-29

**Authors:** Jiqun Wang, Wenwen Liu, Yunyuan Huang, Guangwei Wang, Xiaobo Guo, Donglei Shi, Tianyue Sun, Chaojiang Xiao, Chao Zhang, Bei Jiang, Yuan Guo, Jian Li

**Affiliations:** ^1^ State Key Laboratory of Bioreactor Engineering Shanghai Frontiers Science Center of Optogenetic Techniques for Cell Metabolism Frontiers Science Center for Materiobiology and Dynamic Chemistry Shanghai Key Laboratory of New Drug Design School of Pharmacy East China University of Science and Technology Shanghai 200237 China; ^2^ Key Laboratory of Tropical Biological Resources of Ministry of Education School of Pharmaceutical Sciences Hainan University Haikou 570228 China; ^3^ Hubei Key Laboratory of Genetic Regulation and Integrative Biology School of Life Sciences Central China Normal University Wuhan Hubei 430079 China; ^4^ School of Chemical Engineering Key Laboratory of Synthetic and Natural Functional Molecule of the Ministry of Education Northwest University Xi'an 710127 China; ^5^ Yunnan Key Laboratory of Screening and Research on Anti‐pathogenic Plant Resources from Western Yunnan, Institute of Materia Medica & College of Pharmacy Dali University Dali Yunnan 671000 China; ^6^ Key Laboratory of Xinjiang Phytomedicine Resource and Utilization Ministry of Education School of Pharmacy Shihezi University Shihezi 832003 China

**Keywords:** anti‐aging drugs, combination drugs, salsola collina, senomorphlytic activity

## Abstract

The pursuit of pharmacological interventions in aging aims focuses on maximizing safety and efficacy, prompting an exploration of natural products endowed with inherent medicinal properties. Subsequently, this work establishes a unique library of plant extracts sourced from Yunnan Province, China. Screening of this herbal library herein revealed that *Salsola collina* (JM10001) notably enhances both lifespan and healthspan in *C. elegans*. Further analysis via network pharmacology indicates that the p53 signaling pathway plays a crucial role in mediating the anti‐aging effects of JM10001. Additionally, this work identifies that a composition, designated as JM10101 and comprising three chemical constituents of JM10001, preserves the original lifespan‐extending activity in *C. elegans*. Both JM10001 and JM10101 mitigate aging symptoms in senescence‐accelerated mice treated with doxorubicin and in naturally aged mice. Notably, JM10101 exhibits a more sophisticated senomorphlytic role encompassing both senomorphic and senolytic functions than JM10001 in the modulation of senescent cells, offering a promising strategy for the discovery of combination drugs in the rational development of anti‐aging therapies.

## Graphical abstract

A three‐drug combination discovered in *Salsola collina* for delaying aging phenotypes and extending healthspan is discovered, which can uniquely function as the senomorphlytic drug with both senomorphic and senolytic functions.

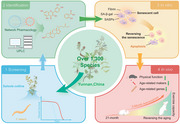

## 1 Introduction

Aging is a complex biological process associated with a spectrum of diseases, including cancer, cardiovascular diseases, Alzheimer's disease, and type 2 diabetes, leading to the deterioration of physiological homeostasis and eventual mortality.^[^ ^1^, ^2^, ^3^ ^]^ Cellular senescence, characterized by cell cycle arrest, apoptosis resistance, and the production of a senescence‐associated secretory phenotype (SASP), occurs in response to various endogenous and exogenous stresses, including DNA damage, telomere dysfunction, and mitochondrial dysfunction. As a result, senescent cells (SnCs) recruit inflammatory cells, remodel the extracellular matrix and damage the normal tissue function, which makes SnCs a reliable target for age‐related disease.^[^ ^4^ ^]^ Researches over the past decades has demonstrated that senotherapeutic strategies can be categorized into senolytic treatments, which clear SnCs, and senomorphic treatments, which reduce SASP,^[^ ^5^, ^6^, ^7^, ^8^ ^]^ both of which are promising approaches to intervene aging and treat age‐related diseases.

Increasing evidence demonstrates that safe and effective drugs, particularly those designed for complex diseases, exert their therapeutic efficacy by interacting with multiple proteins, rather than a single target.^[^ ^9^, ^10^ ^]^ This trend has catalyzed a shift from the traditional one‐drug‐one‐target paradigm to a more holistic multi‐drug‐multi‐target model in the early stages of drug discovery. For thousands of years, natural products have been the primary choice for the prevention and treatment of human diseases in China. These medicines encapsulate profound traditional knowledge and serve as crucial resources for drug development. Chinese herbal medicine (CHM) is characterized by its multi‐component and multi‐target approach, consisting of numerous active compounds that interact with multiple targets.^[^ ^11^, ^12^, ^13^ ^]^ This feature confers a synergistic therapeutic effect to CHM, highlighting its potential in treating complex symptoms that are typically regulated by multiple signaling pathways or mechanisms, such as aging.^[^ ^14^, ^15^ ^]^ Consequently, combination drugs that utilize two or more active agents to enhance clinical outcomes, particularly those derived from CHM, demonstrate superior efficacy compared to mono‐drug therapy in treating complex diseases, reflecting the shared multi‐component, multi‐target feature of CHM.^[^ ^16^ ^]^ Given its superior safety and effectiveness, CHM is increasingly favored in the discovery of combination drugs.^[^ ^17^, ^18^, ^19^ ^]^

**Figure 1:**
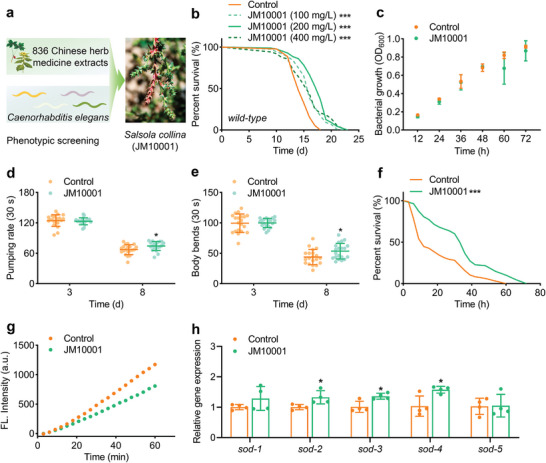
JM10001 extends lifespan and healthspan in WT *C. elegans*. a) The process of screening to obtain JM10001. b) Effects of JM10001 (100, 200, and 400 mg L^−1^) (*n* = 88/87/87) versus the control (*n* = 90) on lifespan. c–f) The effect of JM10001 on bacterial growth (*n* = 20), pumping rate (*n* = 20), body bending (*n* = 20), and survival on paraquat exposure (Control: *n* = 96; JM10001: *n* = 88). g) Worm lysis solution incubated for 1 h with DCFH‐DA in response to ROS levels treated with JM10001 on day 4. h) The mRNA expression of superoxide dismutase genes (*sod‐1*, *sod‐2*, *sod‐3*, *sod‐4*, and *sod‐5*) in N2 worms after JM10001 administration (*n* = 4). The concentration of JM10001 was 200 mg L^−1^ in (c–h). The data were expressed as the mean ± standard deviation. b,f) *P* values were calculated using log‐rank (Mantel‐Cox) test; c,h) *P* values were calculated using two‐sided Student's *t*‐test; d–e) *P* values were calculated using two‐way ANOVA along with Sidak multiple comparisons test (*p < 0.05 and ***p < 0.001).

In this work, we collected 836 Chinese herbal medicine extracts from Yunnan province, southwest China, thereby establishing a unique plant extract library. By evaluating the ability of each plant extract to extend the lifespan in *Caenorhabditis elegans* (*C. elegans*),^[^ ^20^, ^21^ ^]^ we found the *Salsola collina* (JM10001) (**Figure** **1**a). Previously reported pharmacological effects of *Salsola collina* include lowering blood pressure,^[^ ^22^ ^]^ anticancer,^[^ ^23^ ^]^ antioxidant,^[^ ^23^ ^]^ anti‐obesity,^[^ ^24^ ^]^ and lipid‐lowering activities properties,^[^ ^25^ ^]^ with no reports related to anti‐aging. Here, we report its significant anti‐aging effects in *C. elegans* for the first time. Network pharmacology analysis suggested that its anti‐aging effects are associated with the p53 signaling pathway. Furthermore, our innovative approach leverages the fundamental components of CHM to devise unique compositions that enhance their efficacy while minimize the potential toxicity. Consequently, we found that the composition labeled JM10101, consisting of quercetin (Q), β‐sitosterol (β) and salicylic acid (S) derived from JM10001, not only maintained the herb's lifespan‐extending effects both in vitro and in vivo at a lower dose than JM10001, but also exhibited an exciting and unique senomorphlytic (*senomorph*ic & *senolytic*) activity. Taken together, our findings suggest that JM10001 and its derivative composition JM10101 hold potential as anti‐aging agents to delay the aging process and enhance healthspan.

## 2 Results

### 2.1 JM10001 Extends Lifespan and Healthspan in Wild Type *C. elegans*

Our studies commenced with the administration of varying concentrations of JM10001 to WT *C. elegans* (N2) to assess its efficacy in modulating lifespan. Results indicated that our treatment with 100, 200 and 400 mg L^−1^ of JM10001 increased the average lifespan by 9.9%, 19%, and 8.6%, respectively (Figure 1b & Table S1, Supporting Information). Notably, the 200 mg L^−1^ concentration of JM10001 demonstrated the most substantial increase in lifespan, thus it was selected for subsequent experiments. Prior to detailed lifespan studies, we examined whether JM10001 could inhibit the growth of Escherichia coli OP50 (E. coli OP50), the standard diet of *C*. *elegans*, to preclude potential confounding effects of dietary restriction. Growth data for E. coli OP50 treated with JM10001 at various time points were collected using an ultraviolet‐visible spectrophotometer. As shown in Figure 1c, 200 mg L^−1^ JM10001 did not inhibit bacterial growth, confirming that its anti‐aging effects were not caused by dietary restriction. Subsequently, we evaluated the effects of JM10001 on *C*. *elegans* movement behaviors (pharynx pumping and body bending), commonly used metrics to assess healthspan. The results demonstrated that the treatment with JM10001 significantly enhanced the pharyngeal pumping rate and body bending in worms after 8 days (Figure 1d,e). Additionally, the antioxidative capacity is a crucial indicator for evaluating healthspan. Therefore, related experiments including the DPPH assay, survival rate assay in a paraquat‐induced model, DCFH‐DA fluorescent assay, and mRNA expression assay were performed. The DPPH radical scavenging assay is a standard method for assessing the antioxidant activity of compounds in vitro. As shown in Figure S1a, Supporting Information, JM10001 exhibited a dose‐dependent scavenging effect on DPPH radicals across the concentration range of 3.9 to 125.0 mg L^−1^, underscoring its substantial antioxidant activity. Paraquat, an inducer of oxidative stress, was employed to establish a lifespan‐reduced *C*. *elegans* model via the elevation of reactive oxidative species (ROS).^[^ ^26^ ^]^ We further assessed the antioxidant capacity of JM10001 by monitoring the survival rate of *C*. *elegans* in this model. Our findings indicated that the treatment significantly enhanced oxidative stress resistance, resulting in a 62.1% extension of mean lifespan (Figure 1f). Furthermore, we observed dynamic ROS changes in *C. elegans* using the DCFH‐DA fluorescent probe. The results indicated that the JM10001‐treated group exhibited a decrease in ROS fluorescence intensity relative to the control group after 4 days of treatment (Figure 1g, Figure S1b,c, Supporting Information). Furthermore, the mRNA expression levels of superoxide dismutase genes (sod‐2, sod‐3, and sod‐4) was upregulated in the JM10001‐treated group (Figure 1h). Taken together, these findings demonstrate that JM10001 effectively extends the lifespan of *C*. *elegans* and enhances their healthspan.

### 2.2 Key Compounds and Proteins for JM10001's Anti‐Aging

**Figure 2:**
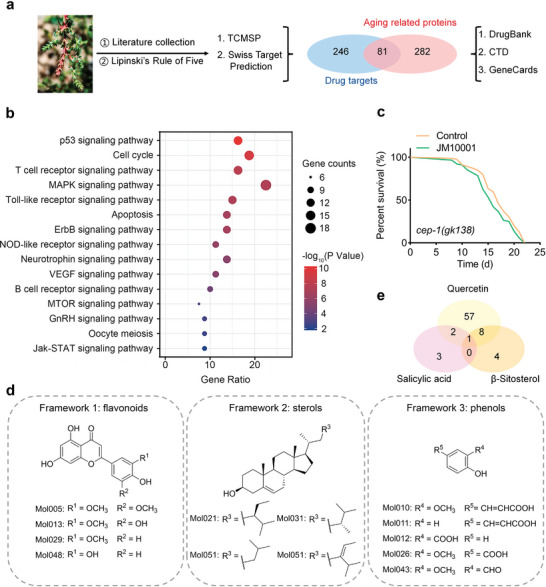
Mechanistic studies and composition discovery. a) Schematic representation of drug and age‐related proteins collection and Venn diagram of their intersection. b) KEGG enrichment analysis of JM10001. c) The lifespan of *cep‐1* mutant worms treated with 200 mg L^−1^ JM10001 (Control: *n* = 79, JM10001: *n* = 88). d) Representative structure framework 1 (flavonoids), framework 2 (sterols), framework 3 (phenols). e) Targets of quercetin, β‐sitosterol, and salicylic acid in common. c) *P* values were calculated using Log‐rank (Mantel‐Cox) test.

To explore the potential anti‐aging mechanisms of JM10001, we utilized a network pharmacology approach, a systems biology method frequently employed to elucidate the overall mechanisms of CHM.^[^ ^27^ ^]^ Initially, we identified 363 age‐related targets from the GeneCards, DrugBank, and CTD databases. Subsequently, 64 ingredients of JM10001 were identified from relevant literature, and 34 potential active compounds were screened using Lipinski's rule of five. Furthermore, we collected 327 potential targets of these active compounds from TCMSP and Swiss Target Prediction databases. To investigate the potential targets of JM10001 in aging, we generated a Venn diagram to identify the overlapping proteins between the targets of JM10001 and age‐related proteins. Consequently, 81 overlapping proteins were obtained and used to construct a protein‐protein interaction network (**Figure** **2**a). Subsequently, these 81 common targets were mapped onto a global protein‐protein interaction network (Figure S2a, Supporting Information). Subsequently, we utilized Cytoscape to visualize the compound‐target network, comprising 101 nodes and 760 edges that include 20 chemical compounds and 81 targets (Figure S2b, Supporting Information). The highest degrees of the topological network were calculated using Cytoscape, which identified Quercetin (Mol048, Q) as the most significant component. To elucidate the potential mechanisms of these 81 key targets, analyses were conducted using the Gene Ontology (GO) and Kyoto Encyclopedia of Genes and Genomes (KEGG) via DAVID database. The GO analysis revealed significant correlations between these targets and biological mechanisms, including regulation of cell proliferation, cell cycle, and apoptosis (Figure S2c, Supporting Information). Furthermore, the KEGG analysis demonstrated that the anti‐aging effect of JM10001 is mediated through various biological pathways, including the p53 signaling pathway, cell cycle, T cell receptor signaling pathway, and MAPK signaling pathway (Figure 2b).

Key targets of JM10001 were primarily concentrated within the p53 signaling pathway, with numerous studies suggesting that p53 may influence age‐related senescence progression^[^ ^28^, ^29^, ^30^ ^]^ Previous studies have demonstrated that cep‐1 in *C*. *elegans*, the ortholog of mammalian p53, gradually increases with aging.^[^ ^31^ ^]^ To identify whether this mechanism contributes to the anti‐aging effects of JM10001, we conducted experiments evaluating the lifespan of cep‐1 mutant worms. As depicted in Figure 2c, JM10001 did not extend the lifespan of cep‐1 mutant worms, indicating that the cep‐1/p53 signaling pathway is integral to its anti‐aging effects. Subsequently, we investigated the effects of JM10001 on cdk‐2, as p53 interacts with the downstream target cyclin‐dependent kinase 2 (cdk‐2), a predicted target whose reduced expression has been shown to prolong the lifespan of *C*. *elegans*.^[^ ^32^ ^]^ Similarly, the result showed that JM10001 did not extend the lifespan of cdk‐2 mutant worms (Figure S2d, Supporting Information), in which the cdk‐2 is the homologous gene of cdk‐2 in mammals. Additionally, we observed a significant reduction in cdk‐2 expression in WT *C. elegans* following administration of JM10001 (Figure S2e, Supporting Information). These results further confirm the critical role of the p53 signaling pathway in the lifespan regulation of JM10001.

The safety and efficacy of CHM have been highly valued in the development of combination treatments.^[^ ^18^, ^33^, ^34^, ^35^ ^]^ These results highlight the significant anti‐aging effects and elucidate the molecular mechanisms of JM10001, facilitating the subsequent discovery of combination drugs. Consequently, we undertook a process to identify potential active components within JM10001. Based on structural similarities, key components of JM10001 were categorized into three groups: flavonoids, sterols, and phenols (Figure 2d). Analysis of component target information revealed that quercetin (Q, Mol048), β‐sitosterol (β, Mol021), and salicylic acid (S, Mol012) exhibited the highest number of targets among those type frameworks (Figure 2e). The combination of Q, β and S encompasses 75 targets, nearly matching the total of 81 targets identified for the active components of JM10001 in the compound‐target network. Subsequently, UHPLC‐Q‐Orbitrap MS/MS confirmed the presence of Q, β, and S in JM10001, using both positive and negative ion modes (Figures S3 and S4, Table S2, Supporting Information). Given these findings, Q, β and S were selected as representative compounds of JM10001 for further studies in combination treatment.

Quercetin, a flavonoid‐derived natural product with diverse bioactivities, has undergone clinical trials in combination with dasatinib for anti‐aging purposes.^[^ ^36^ ^]^ β‐Sitosterol, a phytosterol known for its high safety profile, has been clinically studied for treating prostatic hyperplasia.^[^ ^37^ ^]^ Salicylic acid, a drug clinically approved for the treatment of skin conditions such as psoriasis, demonstrates significant therapeutic efficacy.^[^ ^38^ ^]^ Moreover, JM10001 has a long history of use in traditional Chinese medicine for treating hypertension and headaches, indicating that JM10001 and its three primary components may be safe and exhibit minimal adverse effects.

### 2.3 The Composition JM10101 Extends the Lifespan and Healthspan in *C*. *elegans*

**Figure 3:**
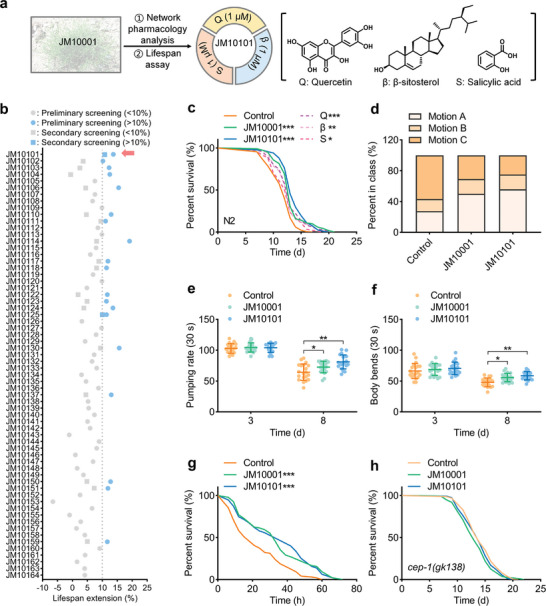
Composition JM10101 extends the lifespan and healthspan in *C. elegans*. a) Flow diagram of anti‐aging compositions from three key compounds, quercetin (Q, Mol048), β‐sitosterol (β, Mol021), salicylic acid (S, Mol012). b) The results of the preliminary and secondary screening. Statistical details of this experiment are summarized in Tables S3 and S4, Supporting Information. c) The lifespan analysis of *C. elegans* treated with JM10001, JM10101, and three compounds (0.30 mg L^−1^ Q, 0.41 mg L^−1^ β, 0.14 mg L^−1^ S), respectively. JM10001 was used as the positive control. Statistical details of this experiment are summarized in Table S1, Supporting Information. d) Effect of JM10101 on the motility of *C. elegans* on day 12. The behavior of worms was classified into Motion A, Motion B, Motion C according to the behavior class. Motion A, youthful and move freely; Motion B, only move slowly after stimulation; Motion C, only move heads or tails after stimulation (*n* = 120). e–g) The effect of JM10101 on pumping rate (*n* = 20), body bending (*n* = 20), and survival on paraquat exposure (Control: *n* = 96; JM10001: *n* = 96; JM10101: *n* = 95). h) The lifespan analysis of *cep‐1* mutant worms with JM10001 and JM10101 (*n* = 159). The concentrations of JM10101 and JM10001 were 0.85 and 200 mg L^−1^ in (b–h), respectively. The data were expressed as the mean ± standard deviation. c,g,h) *p* values were calculated using log‐rank (Mantel‐Cox) test; e,f) *P* values were calculated using two‐way ANOVA along with Sidak multiple comparisons test (*p < 0.05, **p < 0.01, and ***p < 0.001).

To determine the optimal proportion and concentration of the anti‐aging composition Q + β + S (**Figure** **3**a), we conducted high‐throughput lifespan screening. Initially, we evaluated 64 different concentrations of the composition Q + β + S in *C*. *elegans* (Figure 3b), identifying 19 compositions that significantly extended lifespan by more than 10% (Table S3, Supporting Information). Then, these 19 compositions were advanced to secondary screening (Table S4, Supporting Information). As illustrated in Figure 3b, JM10101 demonstrated superior lifespan‐extending activity compared to other compositions. Based on these two rounds of extensive lifespan screening in *C. elegans*, JM10101 (0.85 mg L^−1^, 1 µm Q + 1 µm β + 1 µm S) was chosen for further experimentation.

To explore the potential synergistic effects of JM10101, N2 worms were subjected to various treatments, including JM10101 and its individual components: 0.30 mg L^−1^ Q, 0.41 mg L^−1^ β and 0.14 mg L^−1^ S. As depicted in Figure 3c, the JM10101 group extended the lifespan of the worms by 17.1%, surpassing the increases observed in the Q (8.8%), β (9.5%), and S (8.5%) groups. Of note, JM10101 (17.1%) exhibited a better lifespan extension effect than JM10001 (13.1%) (Table S1, Supporting Information). These results indicate that JM10101 synergistically extends the lifespan of worms. The impact of JM10101 on healthspan was further assessed by monitoring the movement behaviors of *C*. *elegans*. The results suggested that JM10101 significantly enhanced motility, pharynx pumping, and body bending (Figure 3d–f). Furthermore, JM10101 was found to increase antioxidant capacity (Figure 3g) and did not inhibit the growth of OP50 (Figure S5, Supporting Information), ruling out the possibility of dietary restriction interference. Last, we validated the predictive mechanisms of JM10101; the results demonstrated that it did not extend lifespan in cep‐1 or cdk‐2 mutant worms, and it reduced cdk‐2 gene expression in WT worms (Figure 3h, Figure S5b,c, Supporting Information), supporting the cep‐1/p53 signaling pathway as a credible mechanism for its anti‐aging effects. These experimental findings confirm that JM10101 maintains the roles of JM10001 in extending lifespan and enhancing healthspan in *C*. *elegans*.

### 2.4 The Effect of JM10001 and JM10101 on Delaying Senescence in MRC‐5 Cells

**Figure 4:**
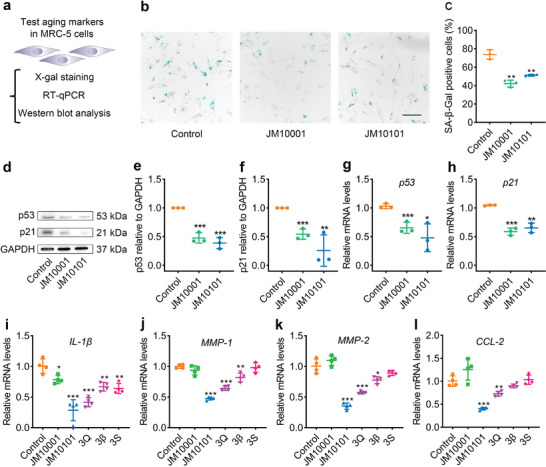
The effect of JM10001 and JM10101 on delaying senescence in MRC‐5 cells. a) Schematic representation of the experiment. MRC‐5 cells were used to evaluate the anti‐aging effect. b) X‐gal staining of MRC‐5 cells and c) quantification of SA‐β‐gal‐positive cells at a late passage (P39) (*n* = 3). Scale bar: 100 µm. The protein levels d–f) and mRNA levels g–h) of p21 and p53 in MRC‐5 cells after JM10001 and JM10101 administration (*n* = 3). i–l) Reverse transcription quantitative polymerase chain reaction (RT‐qPCR) of analysis of SASP factors gene (*IL‐1β*, *MMP‐1*, *MMP‐2*, *CCL‐2*) expression in MRC‐5 cells (P39), GAPDH was used as the loading control (*n* = 4). The concentrations of JM10101 and JM10001 were 6.2 and 50 mg L^−1^ in (c–l), respectively. The concentrations of 3Q, 3β and 3S were 6.6, 9, and 3 mg L^−1^ in (i–l), respectively. The data were expressed as the mean ± standard deviation. (c,e–h,i–l) *p* values were calculated using two‐sided Student's *t*‐test (*p < 0.05, **p < 0.01, and ***p < 0.001).

MRC‐5 cells, human fetal lung fibroblasts, exhibit a typical senescence‐associated phenotype with increasing passage number, making them an ideal model for cellular senescence research.^[^ ^39^, ^40^ ^]^ Consequently, we examined the anti‐aging effects of JM10001 and JM10101 in MRC‐5 cells. Initially, we assessed cell viability and selected JM10001 at 50 mg L^−1^ and JM10101 at 6.2 mg L^−1^, both showing no significant toxicity, to proceed with further experiments (Figure S6a,b, Supporting Information). Cellular senescence is characterized by increased expression of senescence‐associated β‐galactosidase (SA‐β‐gal), p53, p21, and enhanced SASP.^[^ ^41^ ^]^ We thus conducted the X‐gal staining, Western blot, and RT‐qPCR analyses in MRC‐5 cells (**Figure** **4**a). The results indicated a significant reduction in the proportion of SA‐β‐gal positive cells following treatment with JM10001 or JM10101 compared to the control group in late‐passage MRC‐5 cells (P39) (Figure 4b,c). Western blot and RT‐qPCR analyses revealed significant decreases in p53 and p21 expression following JM10001 or JM10101 treatment (Figure 4d–h). Additionally, we conducted immunofluorescence assays to detect γH2AX, a marker of senescence, in MRC‐5 cells and found that JM10001 and JM10101 reduced the foci number of γH2AX (Figure S6c, Supporting Information). Furthermore, compared to JM10001, JM10101 exhibited a superior capacity to reduce the expression of SASP factors, including *IL‐1β*, *MMP‐1*, *MMP‐2*, *CCL‐2*, *IL‐6*, *MIF*, and *CXCL‐2* (Figure 4i–l, Figure S6d–f, Supporting Information). In addition, results also suggest that JM10101 (1Q + 1β + 1S) was more effectively in reducing above SASP factors than the individual higher doses of its components (3Q, 3β, 3S), highlighting its synergistic anti‐aging effects (Figure 4i–l). Collectively, these findings indicate that JM10101 possesses greater potential as a senomorphic agent than JM10001.

**Figure 5:**
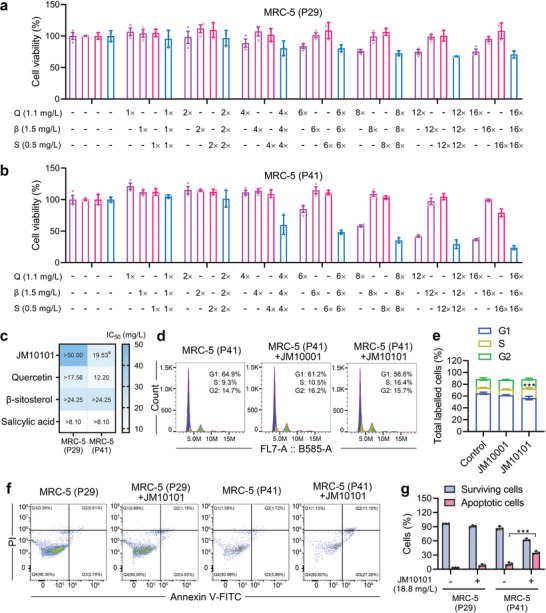
Analysis of JM10101 on aging‐related characteristics in MRC‐5 cells. a–c) Viability of MRC‐5 cells treated with different concentrations of Q, β, S, or JM10101 for 72 h in different passage numbers (P29 and P41) (*n* = 3). The IC50 values of chemical constituents and JM10101 in MRC‐5 cells were depicted. #: 19.5 mg L^−1^: JM10101 = 6.8 mg L^−1^ Q + 9.6 mg L^−1^ β + 3.1 mg L^−1^ S. d,e) Flow cytometry‐based cell cycle analysis of MRC‐5 cells treated with JM10001, and JM10101 for 72 h. The concentrations of JM10101 and JM10001 were 6.2 and 50 mg L^−1^ (*n* = 3). f,g) Representative flow cytometric plots and quantification of the percentage of surviving cells (Q4) and apoptotic cells (Q2 and Q3) in JM10101 (18.8 mg L^−1^)‐loaded MRC‐5 cells (P29 and P41) for 72 h (*n* = 3). The data were expressed as the mean ± standard deviation. e,g) P values were calculated using two‐sided Student's *t*‐test (***p < 0.001).

Given that the composition includes quercetin, a senolytic drug that selectively eliminates SnCs, we examined the SnCs elimination capability of JM10101 and its monomers across various passages of MRC‐5 cells. As demonstrated in **Figure** **5**a,b, with increasing concentration, JM10101 exhibited stronger cytotoxicity in senescent cells at P41 compared to younger cells at P29, confirming its efficacy as a senolytic drug akin to quercetin. However, salicylic acid and β‐sitosterol exhibited minimal cytotoxicity at the tested concentrations in MRC‐5 cells at both P41 and P29. To accurately assess SnCs elimination capability, the half‐maximal inhibitory concentration (IC_50_) was employed to measure cell viability inhibition. IC_50_ values indicated that both JM10101 and quercetin possess enhanced SnCs elimination capabilities compared to β‐sitosterol and salicylic acid (Figure 5c). Notably, 19.5 mg L^−1^ of JM10101, containing 6.8 mg L^−1^ of quercetin, demonstrated equivalent senolytic ability to 12.2 mg L^−1^ of quercetin, suggesting that JM10101 is more effective against senescent cells and outperforms quercetin as a senolytic agent. To further assess the effectiveness of JM10101 as a senolytic agent, we performed experiments on mouse embryonic fibroblasts (MEFs). Our findings showed that P9 MEFs (IC_50_ = 67.1 mg L^−1^) were more sensitive compared to the younger P4 MEFs (IC_50_ = 293.2 mg L^−1^) (Figure S6g,h, Supporting Information), indicating that JM10101 selectively eliminated SnCs in P9 MEFs. To elucidate the mechanism of JM10101 in delaying senescence, we analyzed cell cycle progression and apoptosis following JM10101 treatment using flow cytometry. The results indicated that senescent cells in the 6.2 mg L^−1^ JM10101‐treated group were significantly arrested in the S phase (16.4%) compared to the control group (9.3%), whereas no significant cell cycle arrest was observed at 50 mg L^−1^ JM10001 (Figure 5d,e). Concurrently, flow cytometry analysis demonstrated that the number of apoptotic cells was higher in older P41 MRC‐5 cells compared to younger P29 cells (Figure 5f,g). In general, these results revealed that both JM10001 and JM10101 ameliorated the senescence of MRC‐5 cells, and JM10101 with both senomorphic and senolytic ability exerted a unique senomorphlytic role.

### 2.5 The effect of JM10001 and JM10101 in Mice with Doxorubicin‐Induced Senescence

**Figure 6:**
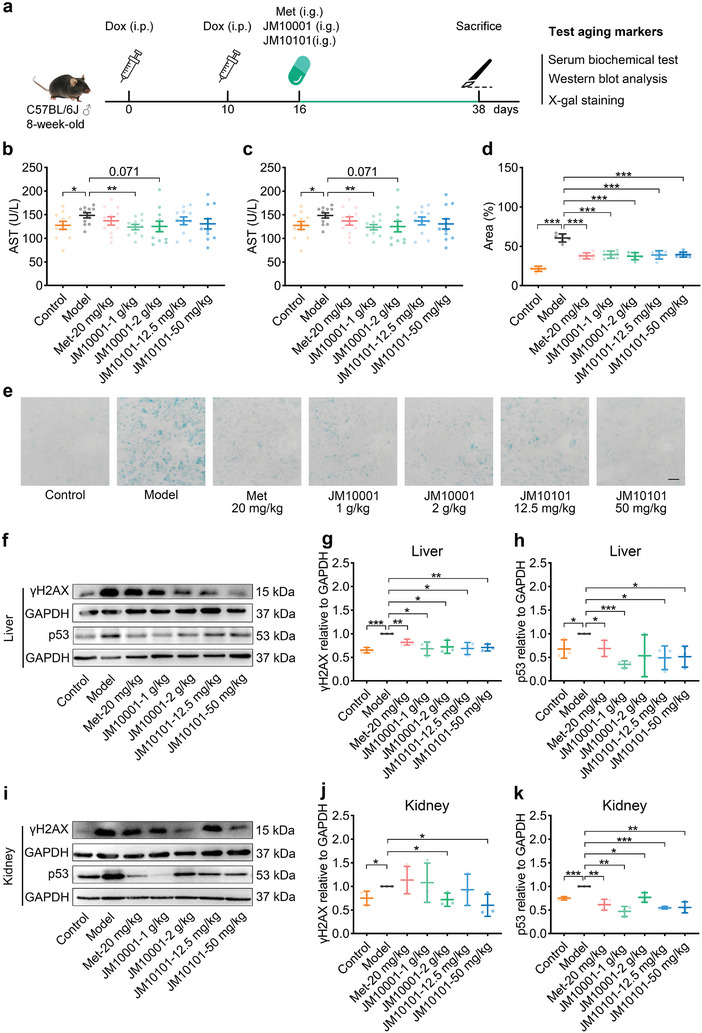
The effect of JM10001 and JM10101 in mice with doxorubicin‐induced senescence. a) Schematic diagram of experimental design for doxorubicin‐induced senescence in mice. b,c) The level of ALT and AST in serum (*n* = 11). d,e) Quantitative area proportion and representative images of SA‐β‐gal stain in kidney sections (*n* = 6). Scale bar: 100 µm. The expression of γH2AX and p53 proteins in f–h) liver and i–k) kidney (*n* = 3). The data were expressed as the mean ± standard deviation. b–d,g.h,j,k) *p* values were calculated using two‐sided Student's *t‐*test (*p < 0.05, **p < 0.01, and ***p < 0.001).

We further explored the anti‐aging effects of JM10001 and JM10101 in mice. Doxorubicin, a chemotherapeutic agent, is known to induce senescence by damaging DNA.^[^ ^42^, ^43^ ^]^ To evaluate the anti‐aging effects of JM10001 and JM10101, we constructed a mouse model with doxorubicin‐induced senescence (**Figure** **6**a, Figure S7, Supporting Information). Specifically, 8‐week‐old C57BL/6J male mice (young control group) received intraperitoneal injections of 5 mg kg^−1^ doxorubicin on day 0 and day 10 to establish the old control group. From day 16 to 37 post‐doxorubicin treatment, mice were divided into five groups, each receiving daily gavage of either 1 g kg^−1^ JM10001, 2 g kg^−1^ JM10001, 12.5 mg kg^−1^ JM10101, 50 mg kg^−1^ JM10101, or metformin (20 mg kg^−1^, as a positive control). From day 16 to 37 post‐doxorubicin treatment, mice were divided into five groups, each receiving daily gavage of either 1 g kg^−1^ JM10001, 2 g kg^−1^ JM10001, 12.5 mg kg^−1^ JM10101, 50 mg kg^−1^ JM10101, or metformin (20 mg kg^−1^, as a positive control). Previous research has shown that doxorubicin significantly increases plasma levels of alanine aminotransferase (ALT) and aspartate aminotransferase (AST), and induces overexpression of SA‐β‐gal in the kidney.^[^ ^44^ ^]^ Our findings indicate that JM10001 and JM10101 effectively counteracted the adverse increases of ALT (Figure 6b) and AST in plasma (Figure 6c), and significantly reduced renal SA‐β‐gal levels (Figure 6d,e). In parallel, we assessed the expression of γH2AX, a protein commonly upregulated in response to DNA damage during the aging process.^[^ ^45^ ^]^ We observed reduced protein levels of both γH2AX and p53 in the liver and kidney (Figure 6f–k), corroborating the results of previous network pharmacology analyses shown in Figure 2. As such, our results demonstrate that JM10001 and JM10101 can effectively counteract drug‐induced senescence in mice.

### 2.6 The Effect of JM10001 and JM10101 in Naturally Aged Mice

**Figure 7:**
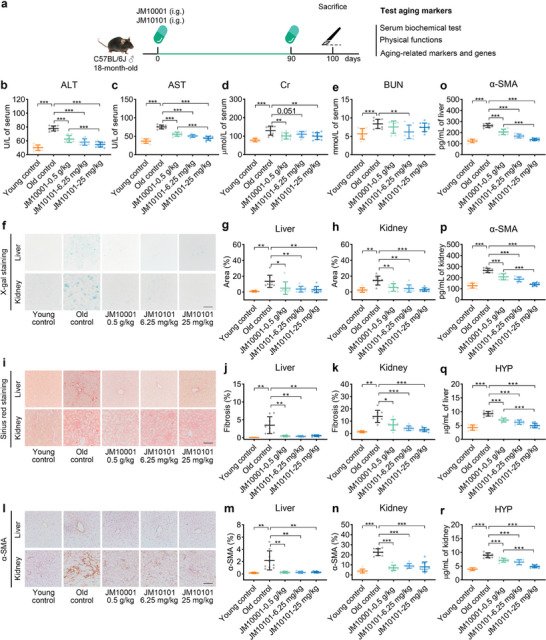
The effect of JM10001 and JM10101 in naturally aged mice. a) Schematic diagram of experimental design for naturally aged mice. b–e) The level of ALT (b), AST (c), Cr (d), and BUN (e) in serum (Young control: *n* = 11; Old control: *n* = 9; 0.5 g kg^−1^ JM10001: *n* = 10; 6.25 mg kg^−1^ JM10101: *n* = 10; 25 mg kg^−1^ JM10101: *n* = 10). f–h) Representative micrographs and quantitative area proportion of SA‐β‐gal stain in liver and kidney sections (*n* = 3). Scale bar: 100 µm. i–k) Representative micrographs and quantitative area proportion of Sirius red stain in liver and kidney sections (*n* = 3). Scale bar: 100 µm. l–n) Representative micrographs and quantitative area proportion of immunohistochemical staining for α‐SMA in liver and kidney sections (*n* = 3). The expression levels of α‐SMA and HYP in o,p) liver and q,r) kidney of mice measured by ELISA (*n* = 9). The data were expressed as the mean ± standard deviation. b–e,g,h,j,k,m,n,o–r) *p* values were calculated using two‐sided Student's *t*‐test (*p < 0.05, **p < 0.01, and ***p < 0.001).

**Figure 8:**
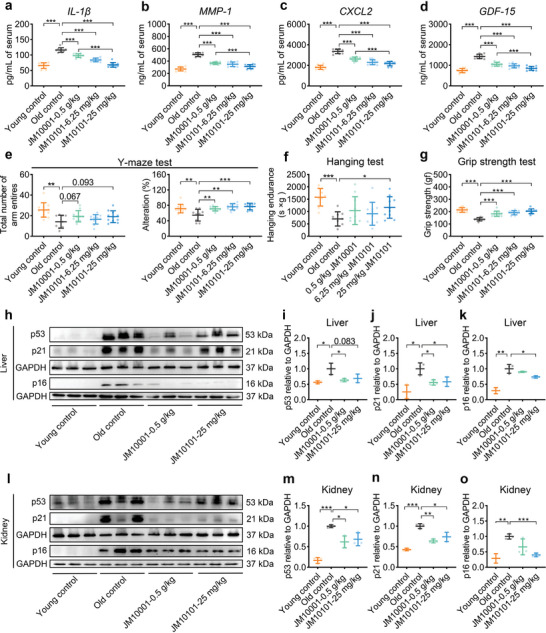
Expression of SASP factors, evaluation of physical function and mechanism validation in naturally aged mice. a–d) The expression of IL‐1β (a), MMP‐1 (b), CXCL2 (c), and GDF‐15 (d) in serum of mice measured by ELISA (*n* = 9). e) Quantification of the total number of arm entires and alteration in Y‐maze test. f) Hanging endurance and g) grip strength of 18‐month‐old male C57BL/6J mice at 3 months after treatment. (Young control: *n* = 11; old control, *n* = 9; 0.5 g kg^−1^ JM10001, *n* = 10; 6.25 mg kg^−1^ JM10101, *n* = 10; 25 mg kg^−1^ JM10101, *n* = 10). The expression of p53, p21, p16 proteins in h–k) liver and l–o) kidney (*n* = 3). The data were expressed as the mean ± standard deviation. a–g,i–k,m–o) *p* values were calculated using two‐sided Student's *t*‐test (*p < 0.05, **p < 0.01, and ***p < 0.001).

To further evaluate the anti‐aging effects of JM10001 and JM10101, we conducted a study involving the treatment of 18‐month‐old mice with these compounds for 3 months. According to the experimental protocol,^[^ ^46^ ^]^ extended treatment periods necessitate dosage reductions. Therefore, the doses of JM10001 and JM10101 were adjusted to 0.5 g kg^−1^ and 25 mg kg^−1^, respectively (**Figure** **7**a, Figure S8, Supporting Information). Subsequently, we measured aging biomarkers in the liver and kidneys of mice across five groups. Our findings revealed that levels of ALT, AST, Cr, and BUN in the serum of aged mice increased significantly but were successfully reduced following administration of either JM10001 or JM10101 (Figure 7b–e). This observation suggests that administering JM10001 and JM10101 can significantly mitigate the decline in liver and kidney function. Experimental results showed that JM10001 and JM10101 significantly reduce the number of senescent cells and fibrosis in the liver and kidneys (Figure 7f–k). Further analyses indicated that JM10001 and JM10101 decreased the levels of α‐smooth muscle actin (α‐SMA) and hydroxyproline (HYP) in the liver and kidneys of naturally aged mice (Figures 7i–r). Additionally, significant improvements in SASP factors were observed in the serum, liver, and kidneys (**Figure** **8**a–d, Figure S9a–h, Supporting Information). Furthermore, naturally aged mice exhibited decreased behavioral responses, which were restored after drug administration (Figure 8e–g). Subsequent mechanistic studies in the liver and kidneys of naturally aged mice confirmed that the anti‐aging effects of JM10001 and JM10101 are mediated through the p53 signaling pathway, which further regulated the levels of p21 and p16 (Figure 8h–o).

To assess the safety of JM10001 and JM10101, we conducted repeated‐dose toxicity experiments in mice. The results indicated no adverse changes in body weight following 14 days of repeated administration of JM10001 (5 g kg^−1^, five times the optimal therapeutic dose) and JM10101 (500 mg kg^−1^, 10 times the optimal therapeutic dose), both administered via gavage (Figure S10a, Supporting Information). Furthermore, no deleterious changes were observed in the relative weights of major organs (heart, liver, spleen, lung, and kidney) after 14 days (Figure S10b, Supporting Information). Examination of serum biochemical markers, including ALT, AST, UREA, and creatinine (Cr) (Figure S10c–f, Supporting Information), showed that JM10001 administration in mice led to decreased ALT levels, which remained within the normal range (mean range, 10.1–96.5 U L^−1^). Additionally, no significant alterations were observed in AST, UREA, and Cr. No significant differences in serum indexes were found between the JM10101 and control groups. Furthermore, histomorphological analysis, using morphology and hematoxylin‐eosin staining, demonstrated no adverse effects on the organs (Figure S11a,b, Supporting Information). Together, these findings confirm the safety profile of JM10001 and JM10101, providing robust evidence supporting their further development as therapeutic agents.

## 3 Discussion and Conclusion

As the global population ages, managing aging becomes increasingly important. Given the complexity of aging, it is imperative to identify therapeutic strategies that are both safer and more effective. Natural products, rich in bioactive compounds, offer a compelling choice for aging management due to their multi‐component, multi‐target, and synergistic therapeutic effects. We collected medicinal plant samples from Yunnan Province located in southwest China, and created a library of 836 herbal extracts to screen for potential agents capable of delaying aging. As a result, we discovered that JM10001, a Chinese herb with various medicinal and edible properties, extends the lifespan of *C. elegans*. Additionally, we observed that JM10001 exerts a protective effect against aging by increasing healthspan and enhancing antioxidative capacity. To further understand the anti‐aging properties of JM10001, we conducted a network pharmacology analysis, which revealed that the p53 signaling pathway plays a role in its anti‐aging activity. We subsequently validated the predicted anti‐aging mechanism through lifespan experiments on *C. elegans* mutants.

Simultaneously, due to similar features between traditional CHM and combination drugs, we categorized the core compounds of JM10001 into three groups: flavonoids, sterols, and phenols. This classification was based on their structural similarities and was conducted using network pharmacology techniques. Quercetin, β‐sitosterol, and salicylic acid were selected based on their extensive target coverage. After evaluating various concentrations of these compounds, the optimal composition (JM10101, 1Q + 1β + 1S) was ultimately identified. We have demonstrated that low concentrations of JM10101 can significantly reduce aging markers such as SA‐β‐gal, p53, p21, and SASP expression at the cellular level. Furthermore, this composition was found to more effectively inhibit the expression of SASP factors compared to each individual monomer. Considering that quercetin is a classical senolytic drug in JM10101,^[^ ^47^ ^]^ we also performed cytotoxicity experiments across various passages of MRC‐5 cells. These studies confirm that JM10101 also exhibits the senolytic effect. Based on these results, and considering the limitations of single‐drug therapy, we propose a strategy to develop senomorphlytics that function both as senolytics to selectively clear senescent cells and as senomorphics to suppress the senescent phenotype through a combination of multiple agents.

To further confirm the anti‐aging effects of JM10001 and JM10101 in vivo, we employed two types of animal models: one with doxorubicin‐induced senescence and the other with natural senescence. Doxorubicin, a prominent chemotherapeutic agent, is frequently implicated in hepatic toxicity. The core mechanism underlying its induction of senescence revolves around the inhibition of DNA topoisomerase II, which subsequently elicits DNA damage and precipitates genomic instability.^[^ ^42^, ^48^ ^]^ Extensive research has consistently demonstrated that premature senescence triggered by doxorubicin is marked by a notable augmentation in key senescence markers, encompassing γH2AX, SA‐β‐gal, and IL‐6, accompanied by elevated levels of hepatic function indicators such as ALT and AST.^[^ ^44^ ^]^ In doxorubicin‐treated mice, JM10001 and JM10101 effectively reduced aging‐related markers and reversed declining liver function. To comprehensively evaluate the anti‐aging effects, we also utilized a naturally aged mouse model. to evaluate anti‐aging effect more comprehensively. The results from X‐gal staining, serum test and pathological staining assays demonstrated that JM10001 and JM10101 mitigated increases in SASP and SA‐β‐gal, as well as the deterioration of renal and liver function in naturally aged mice. Especially, JM10001 and JM10101 countered frailty and enhanced physical function in 21‐month‐old mice. Our animal experiments demonstrated that JM10101 preserves the anti‐aging effects of JM10001, thereby supporting the feasibility of our strategy. A thorough evaluation of the toxicological effects of JM10001 and JM10101 in vivo is essential for their potential clinical applications. Our data indicate that administration of high concentrations of JM10101 (500 mg kg^−1^) did not result in significant systemic toxicity. In contrast, a high concentration of JM10001 (5 g kg^−1^) had a mild effect on liver function in mice, evidenced by reduced ALT levels within the normal range compared to the control group. These findings further demonstrate the superiority and relative safety of JM10101 within our geroprotective strategy.

In summary, our research indicates that JM10001 is a safe and effective traditional CHM with anti‐aging properties, as demonstrated both in vitro and in vivo. Furthermore, we have identified a composition, JM10101, consisting of three chemical constituents derived from JM10001. This composition maintains the original activity of JM10001 in extending the lifespan of *C. elegans* and similarly ameliorates aging markers in mice. Importantly, our in vitro and in vivo findings support that JM10101 enriches and functions as senolytics in SnCs with severe senescence while exists in lower levels and functions as senomorphics in SnCs with less severe senescence, which is more intelligent than JM10001 in regulating SnCs and plays an exciting senomorphlytic role. Consequently, we propose a concept of senomorphlytics (senomorphics & senolytics) as a senotherapeutic strategy, which has landmark significance for the development of anti‐aging drugs.

## 4 Experimental Section

### Preparation of Herbal Extract

JM10001, a plant species authenticated by taxonomists, was collected in Dali (Yunnan, China). The aerial parts of JM10001 (1 kg) were air‐dried and ground into a fine powder. Subsequently, these were subjected to three rounds of extraction using 75% ethanol at 90 °C. The resulting filtrate was concentrated, lyophilized, and stored at −80 °C until further use.

### *C. elegans* Strains, Culture, and Maintenance

Wild type *C. elegans* (N2), TJ1 (*cep‐1(gk138) I*), and VC3074 (*cdk‐2(ok3728) I*) strains were acquired from the *Caenorhabditis* Genetics Center. These strains were cultured at 20 °C on solid Nematode Growth Medium (NGM) agar plates seeded with *E. coli* OP50.

### Lifespan Analysis

Initial synchronization of worm growth involved washing the worms from NGM agar using M9 buffer (1 L water, 5 g NaCl, 6 g Na_2_HPO_4_, 3 g KH_2_PO_4_, and 1 mL 1 mol L^−1^ MgSO_4_). Worm lysis was achieved using a bleaching solution composed of 2.5 mL NaClO solution (13 600–18 400 mg L^−1^ active chlorine), 0.5 mL 10 mol L^−1^ NaOH, and 7 mL water. The lysed worms were then cultured on NGM plates at 20 °C until reaching the L4 stage. Subsequently, age‐synchronized L4 larvae were transferred to NGM plates, with or without test compounds, to determine lifespan. Worms treated with 0.5% DMSO served as the vehicle control. To inhibit progeny development, 50 µg mL^−1^ of 5‐fluorodeoxyuridine was added from day 0 to day 10. Worms were treated with compounds to be tested only 10 days. The number of worms (live or death) was recorded every day and transferred to the fresh plates every 2 days. The survival curves were analyzed using GraphPad Prism 8.

### Bacterial Growth Assay

The assay was performed as previously described.^[^ ^49^ ^]^ 30 µL E. coli OP50 (OD = 0.4) were inoculated onto NGM plates and incubated at 20 °C. The bacteria were completely removed using 1 mL of M9 buffer, and optical density at 600 nm (OD_600_) was measured using a Hitachi U‐2910 spectrometer at designated time points (12, 24, 36, 48, 60, and 72 h). M9 buffer served as the blank control.

### Pharyngeal Pumping Assay and Thrashing Assay

Pharyngeal pumping assay and thrashing assay were performed as previously described.^[^ ^50^ ^]^ To calculate the pharyngeal pumping rate, 20 worms were individually placed on plates seeded with OP50 and left undisturbed for at least 20 min, with counts conducted on days 3 and 8. Pharyngeal pumps were recorded for 30 s, counting every second pump; these values were then doubled to calculate the total number of pumps. For the body bend analysis, M9 buffer was dropped onto the blank NGM plate, and each worm was allowed 30 s to acclimate. The number of complete body swings was then counted for each worm. The number of complete body swings was then counted for each worm over a 30‐s period on days 3 and 8. Any change in the direction of the bend in the midsection of the body was defined as a thrash.^[^ ^51^ ^]^

### Oxidative Stress Resistance Assay

This assay was performed as previously described.^[^ ^52^, ^53^ ^]^ Following a 4‐day treatment, 90 worms were allocated to each experimental group. Worms were then transferred to 24‐well plates containing 10 mM paraquat. The number of surviving worms was counted at 2‐h intervals until all the worms were dead.

### Motility Assay

This assay was conducted as previously described.^[^ ^54^ ^]^ A minimum of 90 worms were prepared for each group and subjected to a 12‐day treatment period. Locomotor phenotypes were assessed through gentle tactile stimulation under stereo‐microscope. Worms exhibiting Motion A were classified as youthful and demonstrated free movement. Worms that exhibited only slow head or tail movement upon stimulation were categorized as Motion C. Motion B was classified as an intermediate state between Motion A and Motion C.

### 2,2‐Diphenyl‐1‐Picrylhydrazyl Radical Scavenging Assay

The antioxidant capacity of the compounds was evaluated using the DPPH radical scavenging assay, as previously described.^[^ ^55^ ^]^ In brief, 100 µL of a 200 µm DPPH solution was combined with 100 µL of JM10001 at various concentrations in 96‐well plates. Ethanol replaced DPPH as the blank control. The mixture was then incubated at 37 °C for 30 min, and the absorbance was measured at 517 nm. The percentage of DPPH radical scavenging was calculated using the formula: [(A_0_ – A_1_)/A_0_] × 100. A_0_ is the absorbance of the control and A_1_ is the absorbance of the JM10001.

### Measurement of ROS Level

ROS levels were quantified using DCFH‐DA (Yeasen) following previously established methods, with some modifications.^[^ ^56^ ^]^ ≈1500 *C*. *elegans* (day 4) were removed from plates using M9 buffer, transferred to tubes, and washed thrice to eliminate bacteria. Subsequently, the worms were transferred to PBS and immediately flash‐frozen in liquid nitrogen for 10 min. The worms were then thawed at room temperature and lysed via sonication (UXI, JY92‐IIN) for 6 min on ice, followed by centrifugation at 13 000 rpm for 30 min at 4 °C. The supernatant was collected and its protein content quantified using a BCA assay (Yeasen). Then, 50 µg protein solution was then incubated with 200 µL DCFH‐DA (250 µm) at 37 °C for 1 h, and fluorescence was measured using a fluorospectro photometer (Techcomp, F‐4700). Ultimately, ROS levels were proportional to the measured fluorescence intensity.

### Collection of Information on Anti‐Aging Related Proteins and JM10001 Ingredients

Anti‐aging related proteins were sourced from CTD, DrugBank and GeneCards databases.^[^ ^57^, ^58^, ^59^ ^]^ Active ingredients were identified from the scientific literature. The term “Salsola collina” was utilized as a search keyword in two databases: CNKI and SciFinder. Chemical structures were retrieved from Pubchem (https://pubchem.ncbi.nlm.nih.gov/). The targets for active ingredients were retrieved using the TCMSP (http://tcmspw.com/tcmsp.php) and Swiss Target Prediction (http://swisstargetprediction.ch/) databases.^[^ ^60^, ^61^ ^]^ SMILES numbers of each component were entered into the Swiss Target Prediction to collect targets with a probability of ≥ 0.3. Age‐related genes and JM10001 active ingredient targets, gathered from the databases, were compared using VENNY (https://bioinfogp.cnb.csic.es/tools/venny/) to identify common targets between JM10001 and aging.

### Network Construction and Analysis

Targets obtained were inputted into STRING (https://string‐db.org/) to construct Protein‐Protein Interaction (PPI) networks. The species was defined as “Homo sapiens,” and the highest confidence level was set at 0.7. Based on key targets identified from the Venn diagram, Cytoscape 3.6.0 was employed to construct a network of interactions between active compounds and these targets.^[^ ^62^ ^]^

### Gene Ontology Enrichment Analysis and Kyoto Encyclopedia of Genes and Genomes Pathway Analysis

GO enrichment and KEGG pathway analyses were performed using DAVID (https://david.ncifcrf.gov/) to identify relevant functions and pathways associated with key targets.

### Cell Culture and Cytotoxicity Assay

MRC‐5 cells were kindly provided by Stem Cell Bank, Chinese Academy of Sciences. MRC‐5 cells were cultured in MEM (Gibco) supplemented with 1% gluta‐max (Gibco), 1% non‐essential amino acids (Gibco), 1% sodium pyruvate (Gibco), 1% penicillin/streptomycin (Yeasen) and 10% fetal bovine serum (Gibco). MRC‐5 cells were maintained at 37 °C under 5% CO_2_. MEFs were isolated from E13‐E14 embryos following previously described methods.^[^ ^63^ ^]^ Embryonic tissue from donor mice was minced and digested with 0.05% trypsin‐EDTA (Gibco), containing 100 Kunitz units of DNase I, for 15 min at 37 °C. Cells were cultured in 0.2% gelatin‐coated dishes using high‐glucose DMEM (Gibco) supplemented with 10% fetal bovine serum and 1% penicillin/streptomycin. For the cytotoxicity assay, 8000 MRC‐5 or MEFs cells per well were seeded in a‐96‐well plate and treated with compounds for 72 h. For CCK‐8 detection, 10 µL of CCK‐8 reagent (Targetmol) was added to each well and incubated for 2 h prior to analysis. Absorbance at 450 nm was measured using a Microplate Reader (Bio‐Tek Instruments, Synergy, H1).

### SA‐β‐gal Staining Assay

The SA‐β‐gal staining assay was performed using an SA‐β‐gal staining kit (Beyotime), following the manufacturer's instructions, to evaluate SA‐β‐gal content in MRC‐5 cells, MEFs cells and mouse kidneys. MRC‐5 or MEFs cells were seeded at a density of 10 000 cells per well in 1000 µL of medium in 24‐well plates and treated with the compound of interest for 72 h. MRC‐5 cells were seeded in 24‐well plates with 10 000 cells per well in 1000 µL medium, and treated with related compound for 72 h. Subsequently, the cells were washed three times with PBS and then fixed in fixative solution for 15 min at room temperature. A fresh staining solution was then applied to the fixed cells, which were incubated at 37 °C overnight. The next day, the cells were washed three times with PBS and stained with DAPI to quantitatively assess SA‐β‐gal activity. Animal kidneys were embedded in Optimal Cutting Temperature (OCT) compound (Leica), sectioned at 20 µm using a cryocut microtome (CRYOSTAR NX50, Thermo), and mounted on slides (Servicebio). The staining protocol for the kidneys mirrored that used for the cells as described above. Imaging was captured using a Nikon Eclipse Ts2R inverted microscope.

### Quantitative Real‐Time PCR

Samples from of N2 worms and MRC‐5 cells were prepared, and RNA was extracted. Total RNA was extracted using the Total RNA Kit (OMEGA, bio tec, R6934‐01). Reverse transcription was performed using the reverse transcription kit (Yeasen, 11141ES60). qPCR was carried out using the Hieff UNICON Universal Blue qPCR SYBR Green Master Mix (Yeasen, 11184ES08). Primers for qPCR are listed in Table S5, Supporting Information.

### Western Blot

RIPA lysis buffer (Yeasen, 20114ES60 for cells and 20101ES60 for tissues) was used to extract proteins form cells, liver, and kidney tissues. Protein concentrations were determined using the BCA Protein Assay Kit (Yeasen, 20201ES76). Proteins were separated using SDS‐PAGE (12% or 13.5%) and transferred onto an NC membrane, which was blocked with 5% milk in TBST for 2 h. Membranes were incubated overnight at 4 °C with primary antibody (p53: Santa Cruz Biotechnology, sc‐126, 1/500 dilution; p21: Santa Cruz Biotechnology, sc‐6246, 1/500 dilution; γH2AX: Abcam, ab81299,1/10 000 dilution; p16: Abcam, ab211542, 1/2000 dilution; GADPH: Proteintech, 60004‐1‐Ig, 1/100 000 dilution). Following 3 washes with TBST, membranes were incubated for 1 h at room temperature with secondary antibody (goat anti‐rabbit IgG anti‐body: Yeasen, 33101ES60, 1/100 000 dilution; goat anti‐mouse IgG anti‐body: Yeasen, 33201ES60, 1/100 000 dilution). After additional washes with TBST, the membranes were incubated with Super ECL Detection Reagent (Yeasen, 36208ES76). Imaging was performed using the Tanon‐4600SF system, and images were analyzed with ImageJ software.

### Immunofluorescence Staining

Cells for immunofluorescence staining assays were incubated with primary antibody (γH2AX: Abcam, ab81299,1/200 dilution) at 4 °C overnight. After that, cells were washed for three times with PBS and then incubated with secondary antibodies (anti‐rabbit IgG antibody, CST, 7074P2, 1/500 dilution) for 1 h at 37 °C. The samples were then stained by Hoechst.

### Flow Cytometric Assay

For cell cycle assay, MRC‐5 cells (P41) were seeded in six‐well plates and allowed to adhere adhered for 24 h, followed by synchronization in serum‐free medium for 12 h. Cells were then treated with JM10001 (50 mg L^−1^) and JM10101 (6.2 mg L^−1^) for 72 h. After trypsinization, cells were collected, rinsed with PBS, fixed in 70% ethanol at 4 °C overnight, and processed according to the Cell Cycle and Apoptosis Analysis Kit (Beyotime, C1052). For apoptosis analysis, MRC‐5 cells (P29 and P41) were seeded in six‐well plates and cultured for 24 h, and then treated with JM10101 (18.8 mg L^−1^) for 72 h. Subsequently, cells were washed with PBS and stained following the Annexin‐FITC/PI Apoptosis Detection Kit protocol (Elabscience, E‐CK‐A211). Samples from the both assays were analyzed using a flow cytometer (CytoFLEX LX, Beckman Coulter). Data were analyzed using FlowJo software, and statistical analysis was performed using GraphPad Prism 8.0.

### UHPLC‐Q‐Orbitrap MS/MS

An Agilent ZORBAX Eclipse Plus C18 column (100 mm × 2.1 mm, 1.8 µm) was used in this experiment. The mobile phase comprised 0.1% (v/v) formic acid in water and acetonitrile, with a flow rate of 0.3 mL min^−1^ and an injection volume was 5 µL. The gradient program was set as follows: 0–20 min at 10% to 50% acetonitrile; 20–25 min at 50% to 90% acetonitrile; 30–30.01 min at 90% to 10% acetonitrile; 30.01–35 min, 10% acetonitrile. MS/MS analysis was conducted in both ESI positive and negative ion modes. The temperature of the capillary and aux gas heater were set at 350 and 250 °C, respectively. The aux gas flow rate was 10 L h^−1^, and the spray voltage was 3200 V. Positive ions were scanned over an m/z range from 150 to 2000, and negative ions from 100 to 1400. High‐resolution mass spectra were obtained using a Thermo Scientific Exactive Plus mass spectrometer (Thermo Scientific, USA). Data acquisition was performed using Xcalibur software.

### Mice Assay

Male C57BL/6J mice aged 8–12 weeks and 18 months were acquired from Charles River (Beijing, China) and Wukong Biotechnology (Jiangsu, China). All animals were maintained under specific pathogen‐free conditions and experiments were conducted in accordance with National Institutes of Health guidelines. The mice assays were approved by the Institutional Animal Care and Use Committee of Hainan University, in compliance with Chinese laws for experimental animals (approval number SYXK (Hainan) 2023‐0031). Mice experiments were performed as previously described.^[^ ^43^, ^64^ ^]^ To establish a mouse model of doxorubicin‐induced senescence, mice were intraperitoneally injected with 5 mg kg^−1^ doxorubicin on day 0 and day 10. Metformin, JM10001, and JM10101 were administered through oral gavage from day 15 to 37 using a vehicle composed of 20% corn oil and 80% saline. Serum form mice with doxorubicin‐induced senescence were collected by centrifuging blood (10 000 g at 4 °C for 30 min). In naturally aged mice model, JM10001, and JM10101 were administered through oral gavage from day 0 to 90 using a vehicle composed of 20% corn oil and 80% saline. In a naturally aged mouse model, JM10001 and JM10101 were administered via oral gavage from day 0 to day 90 using a vehicle composed of 20% corn oil and 80% saline. Serum, liver, and kidney samples form naturally aged mouse model were sent to the Ruifan Biological Technology (Shanghai, China) for analysis using ELISA kit (ALT: RF8547, AST: RF8502, Cr: RF8275 BUN: RF8274, IL‐1β: RF7630, MMP‐1: RF7679, CXCL2: RF8696, GDF‐15: RF7194, α‐SMA: RF8543, HYP: RF7898). ELISA data collection and analysis were performed by the company.

### Sirius Red Staining

Liver and kidney tissues were harvested and fixed in 4% paraformaldehyde. Following paraffin embedding, 4 µm thick sections were cut, deparaffinized, and stained with Sirius red. Images were captured with a Nikon Eclipse Ts2R inverted microscope, and the positive areas were quantified using ImageJ software.

### Immunohistochemical Staining

After deparaffinization, liver and kidney sections were treated with endogenous peroxidase blocking buffer for 25 min, then incubated with serum (Solarbio, SL034, 1/20 dilution) at room temperature for 30 min. The sections were incubated overnight with the primary antibody (α‐SMA, abcam, ab124964, 1/5000 dilution) at 4 °C, then with the secondary antibody (HRP‐linked goat anti‐rabbit IgG, ZSGB‐BIO, PV‐6001, 1/2000 dilution) for 50 min at room temperature. After developing with diaminobenzidine peroxidase substrate, nuclei were counterstained with hematoxylin and eosin. Images were captured using a Nikon Eclipse Ts2R inverted microscope, and positive areas were quantified using ImageJ software.

### Physical Function Measurements

The experimental procedures were based on methods described in previously published papers.^[^ ^40^, ^65^ ^]^ All measurements were conducted at 5 days after the final treatment. Spatial working memory was assessed using a Y‐shaped maze (Shanghai XinRuan Information Technology, XR‐XY1032). Mice were individually placed into one of the three arms of the maze and allowed to explore. Total arm entries and the spontaneous alternation were recorded and analyzed over an 8‐min period. The hanging test was performed using 2‐mm‐thick metal wire, positioned 35 cm above a padded surface. Mice were allowed to grab the wire with forelimbs only, and the hanging time was then recorded. Hanging endurance was normalized to body weight, calculated as hanging time (s) multiplied by body weight (g). Each mouse was given three trials, and the average hanging time was calculated. For the grip strength test, mice were positioned on the grid of a grip strength meter (Shanghai XinRuan Information Technology, XR501) and grasped with their forelimbs. Each mouse was gently pulled backward by the tail until it released its grip, and the maximum grip strength form six tests was recorded.

### Repeat‐Dose Toxicity Studies

Male C57BL/6J mice, 8 weeks old, were sourced from Charles River (Beijing, China). Mice experiments were performed as previously described.^[^ ^66^ ^]^ Mice were randomized into three groups (n = 8, males and females), including the control group (vehicle: 20% corn oil and 80% saline), JM10001‐treated group (5 g kg^−1^, i.g.), and JM10101‐treated group (500 mg kg^−1^, i.g.). Mice were administered treatments via gavage each morning; body weights were recorded and toxicity symptoms were monitored over a 14‐day period. At the end of the experimental period, mice were sacrificed, major organs (heart, liver, spleen, lungs, and kidneys) were dissected and macroscopically evaluated to calculate relative weights. Serum was collected for biochemical analysis, and major organs for Hematoxylin and eosin (HE) staining (Servicebio).

### Statistical Analysis

Data form Figure 1h; Figure 4e–h,i‐l; Figure 5a,b; Figure 6g,h,j,k; Figure 8i–k,m–o were preprocessed and normalized to the controls. ImageJ was used to process imaging data. Data analysis was performed using GraphPad Prism and FlowJo. The data were expressed as the mean ± standard deviation. Statistical analyses were performed in GraphPad Prism with log‐rank (Man‐Cox) test, two‐sided Student's t‐test and two‐way ANOVA along with the Sidak multiple comparisons test. The sample size (n), reported P values, and statistical tests are reported in relevant figure legends. Significance was defined as *p < 0.05, **p < 0.01 and ***p < 0.001. In the in vivo experiments, mice were randomly assigned to treatment groups. No animals or samples were excluded from the analyses. The studies were not performed blindly, and all data collection and analysis were objective in nature.

## Conflict of Interest

The authors declare no conflicts of interest.

## Author Contributions

J.Q.W., W.W.L., and Y.Y.H. contributed equally to this work. Y.G. and J.L. conceived and designed the project. Y.G., J.L., Y.Y.H., W.W.L., J.Q.W., D.L.S., and G.W.W. wrote and revised the manuscript. J.Q.W., X.B.G., T.Y.S. performed and analyzed the experiments. B.J., C.J.X. and C.Z. provided research materials.

## Supporting information

Supporting Information

## Data Availability

The data that support the findings of this study are available from the corresponding author upon reasonable request.
