# Correction to “A Senomorphlytic Three‐Drug Combination Discovered in *Salsola collina* for Delaying Aging Phenotypes and Extending Healthspan”

**DOI:** 10.1002/advs.202505321

**Published:** 2025-04-25

Jiqun Wang, Wenwen Liu, Yunyuan Huang, Guangwei Wang, Xiaobo Guo, Donglei Shi, Tianyue Sun, Chaojiang Xiao, Chao Zhang, Bei Jiang, Yuan Guo, and Jian Li. A Senomorphlytic Three‐Drug Combination Discovered in *Salsola collina* for Delaying Aging Phenotypes and Extending Healthspan. *Adv. Sci*. **2024**, *11*, 2401862. https://doi.org/10.1002/advs.202401862

Theimage depicting serum ALT test results shown in Figure 6b was incorrect.


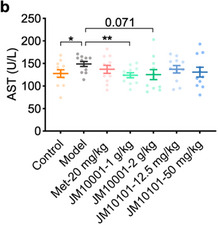


This should have read:


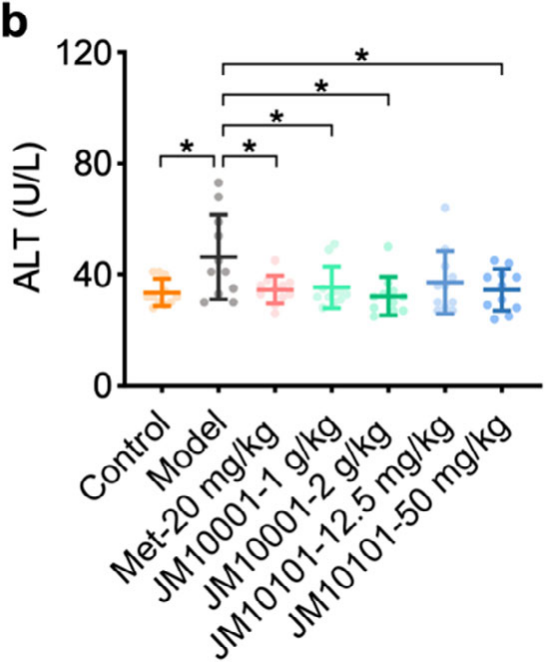


We apologize for mistakenly placing the AST image here.

## Supporting information



Supporting Information

