# Supplementary material for: A Senomorphlytic Three‐Drug Combination Discovered in Salsola collina for Delaying Aging Phenotypes and Extending Healthspan
Source: Adv Sci (Weinh). 2024 Jul 29;11(36):2401862. doi: 10.1002/advs.202401862 (PMC11423240; doi:10.1002/advs.202401862)
Supplement: Supplementary file 1 — Supporting Information [file ADVS-11-2401862-s001.doc]

**Supporting Information**

**A Senomorphlytic Three-drug Combination Discovered in *Salsola collina* for Delaying Aging Phenotypes and Extending Healthspan**

*Jiqun* *Wang ^1^, Wenwen Liu ^3^, Yunyuan Huang ^4^, Guangwei Wang ^2^, Xiaobo Guo ^1^, Donglei Shi ^3^, Tianyue Sun ^1^, Chaojiang Xiao ^5^, Chao Zhang ^1^, Bei Jiang ^*5^, Yuan Guo ^*2^ & Jian Li ^*1,3,6^*

^1^State Key Laboratory of Bioreactor Engineering, Shanghai Frontiers Science Center of Optogenetic Techniques for Cell Metabolism, Frontiers Science Center for Materiobiology and Dynamic Chemistry, Shanghai Key Laboratory of New Drug Design, School of Pharmacy, East China University of Science and Technology, Shanghai 200237, China.

^2^School of Chemical Engineering, Key Laboratory of Synthetic and Natural Functional Molecule of the Ministry of Education, Northwest University, Xi’an 710127, China.

^3^Key Laboratory of Tropical Biological Resources of Ministry of Education, School of Pharmaceutical Sciences, Hainan University, Haikou 570228, China.

^4^Hubei Key Laboratory of Genetic Regulation and Integrative Biology, School of Life Sciences, Central China Normal University, Wuhan, Hubei 430079, China.

^5^Yunnan Key Laboratory of Screening and Research on Anti-pathogenic Plant Resources from Western Yunnan, Institute of Materia Medica & College of Pharmacy, Dali University, Dali, Yunnan 671000, China.

^6^Key Laboratory of Xinjiang Phytomedicine Resource and Utilization, Ministry of Education, School of Pharmacy, Shihezi University, Shihezi 832003, China.

Jiqun Wang, Wenwen Liu, Yunyuan Huang contributed equally to this work.

*Corresponding authors: Jian Li (jianli@ecust.edu.cn), Yuan Guo (guoyuan@nwu.edu.cn), Bei Jiang (jiangbei@dali.edu.cn)

**Table of Contents**

**Supplementary Figures** S3

Figure S1. S3

Figure S2. S3

Figure S3. S4

Figure S4. S5

Figure S5. S6

Figure S6. S7

Figure S7. S8

Figure S8. S8

Figure S9. S8

Figure S10. S9

Figure S11. S10

**Supplementary Tables** S11

Table S1 S11

Table S2 S12

Table S3 S12

Table S4 S15

Table S5 S16

**Supplementary Figures**


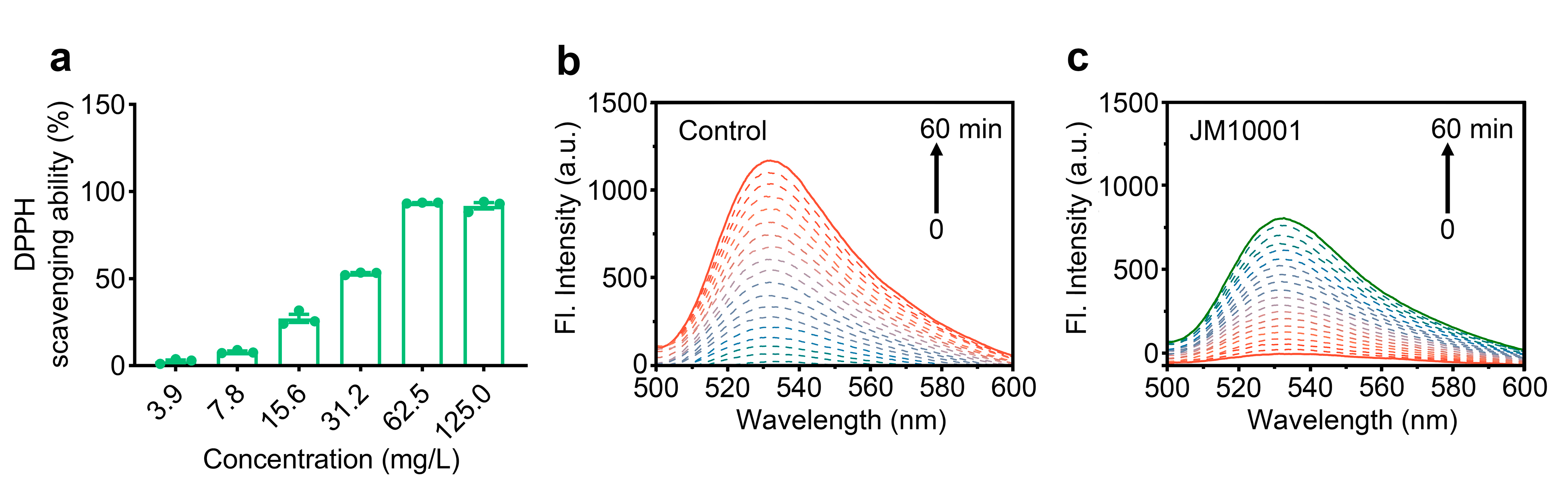


**Figure S1.** Antioxidant capacity of JM10001 *in* *vitro* and *in* *vivo*. (a) The DPPH radical scavenging ability of JM10001 (*n* = 3). (b-c) Worm lysis solution was incubated for 1 hour with DCFH-DA staining in response to ROS levels. The data were expressed as the mean ± standard deviation.


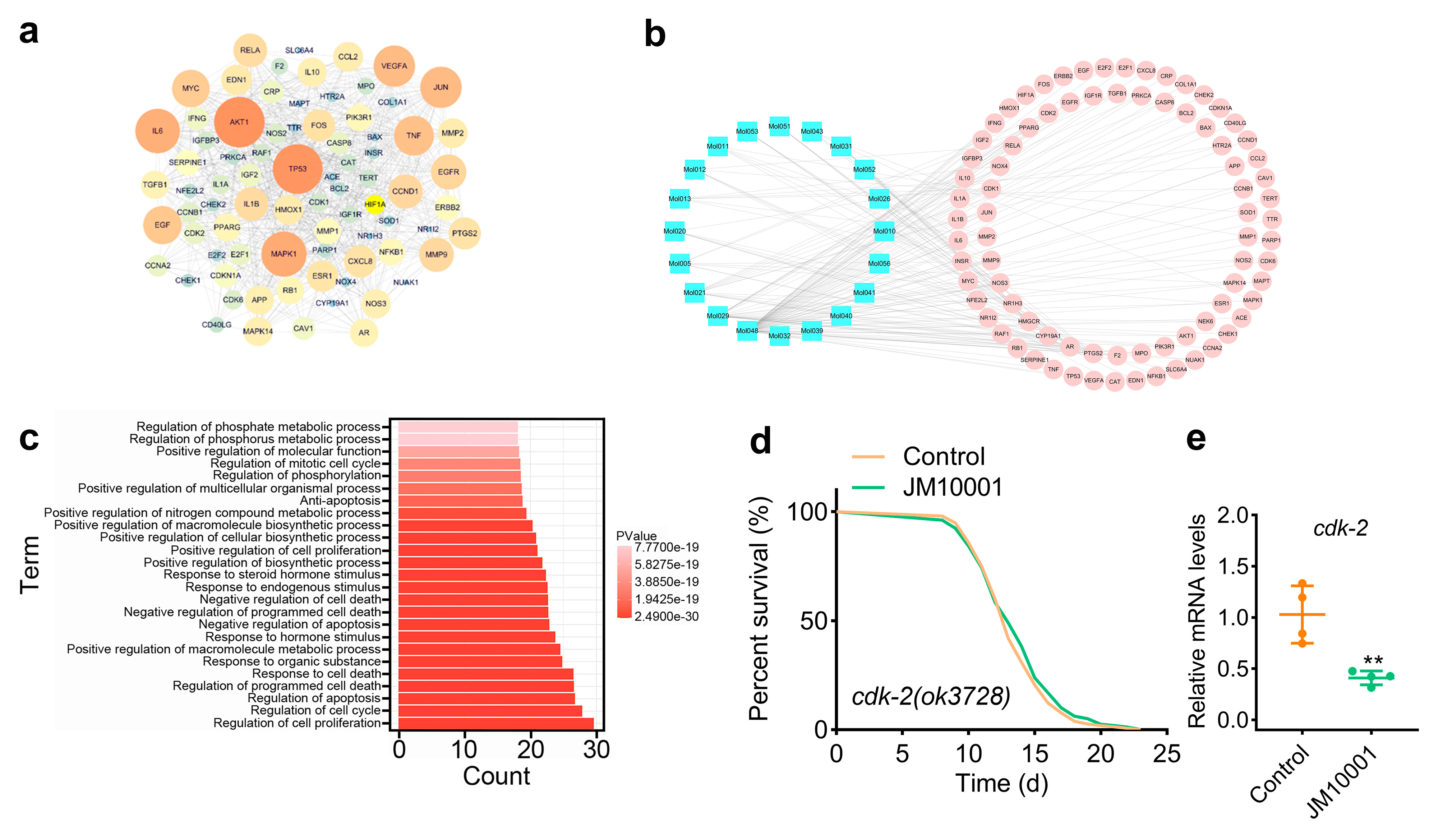


**Figure S2.** Network pharmacological analysis of JM10001. (a) The protein-protein interaction networks were constructed by STRING from the intersection of drug targets and age-related proteins. (b) Compounds-targets network (blue squares indicate compounds and red circles indicate targets). (c) GO enrichment analysis. (d) The lifespan of *cdk-2* mutant worms treated with 200 mg/L JM10001 (Control: *n* = 156; JM10001 *n* = 158). (e) The mRNA level of *cdk-2* in WT *C. elegans* (*n* = 4). The data were expressed as the mean ± standard deviation. (d) *P* values were calculated using log-rank (Mantel-Cox) test; (e) *P* values were calculated using two-sided Student’s t-test (***P* < 0.01).


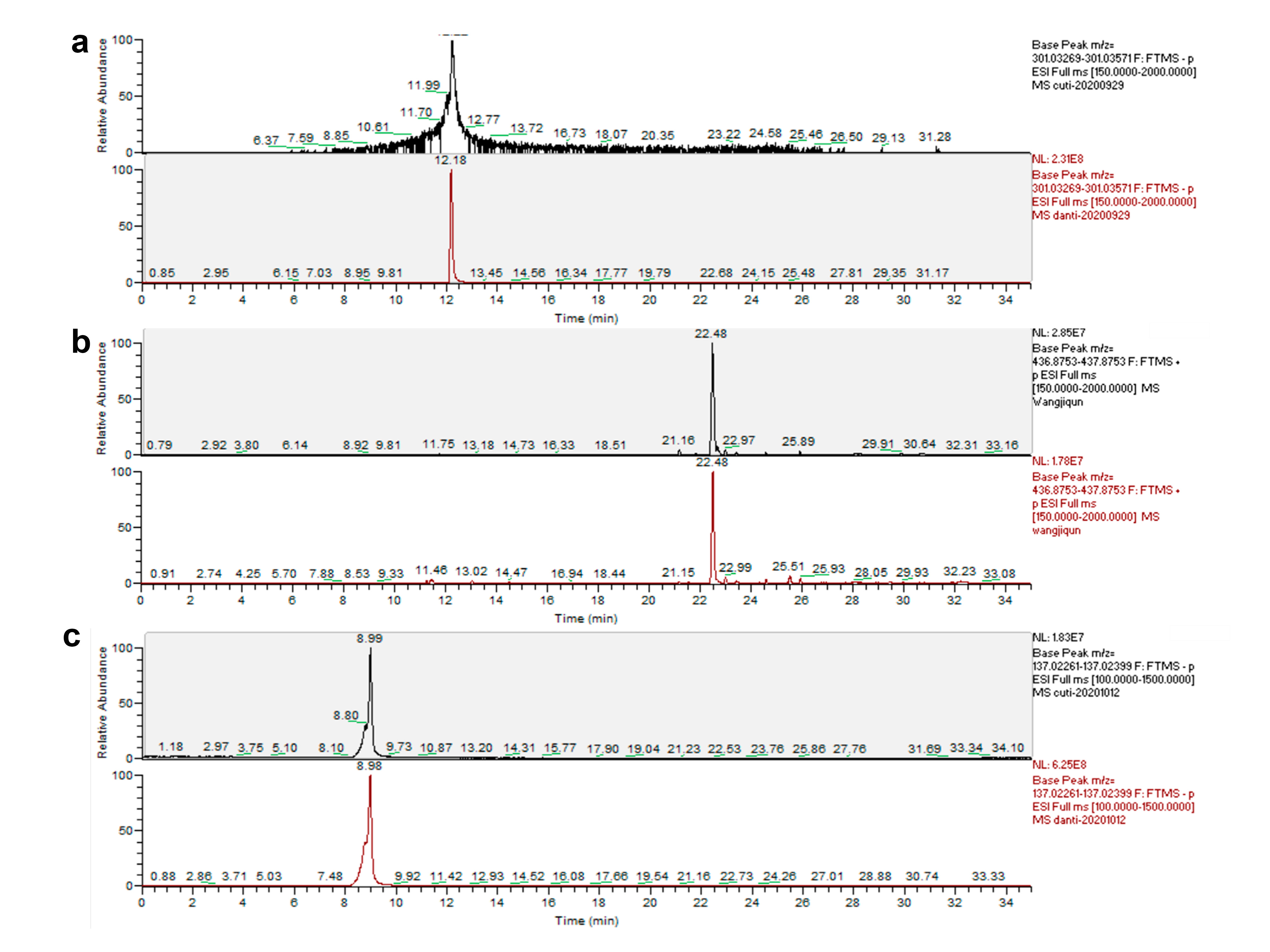


**Figure S3.** UPLC chromatograms of JM10001 and single ingredient. (a) quercetin, (b) β-sitosterol, (c) salicylic acid. The top half of each image is JM10001 and the bottom half is a single ingredient.


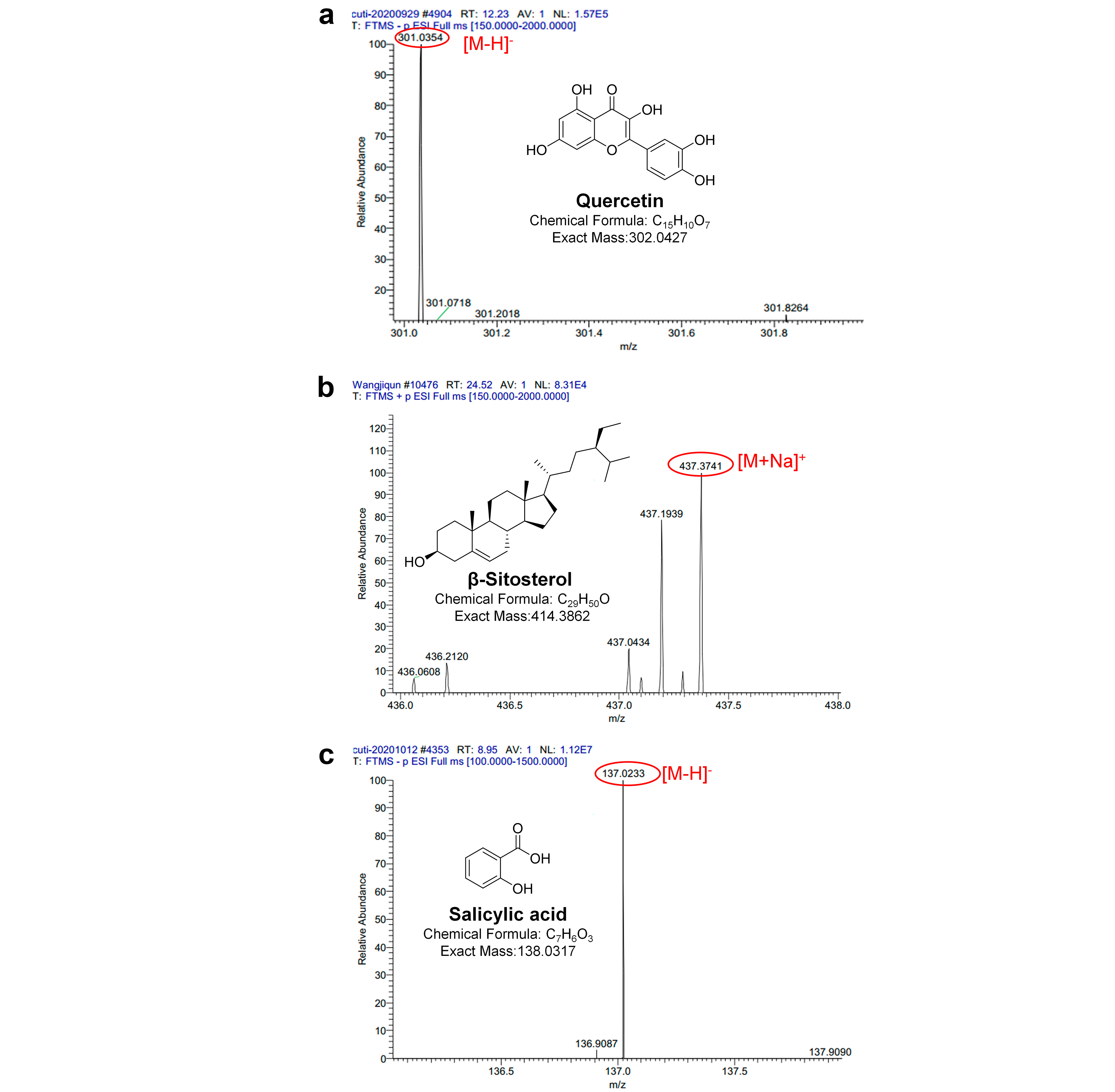


**Figure S4.** High resolution mass spectrometer of a single ingredient in JM10001 (a) quercetin, (b) β-sitosterol, (c) salicylic acid.


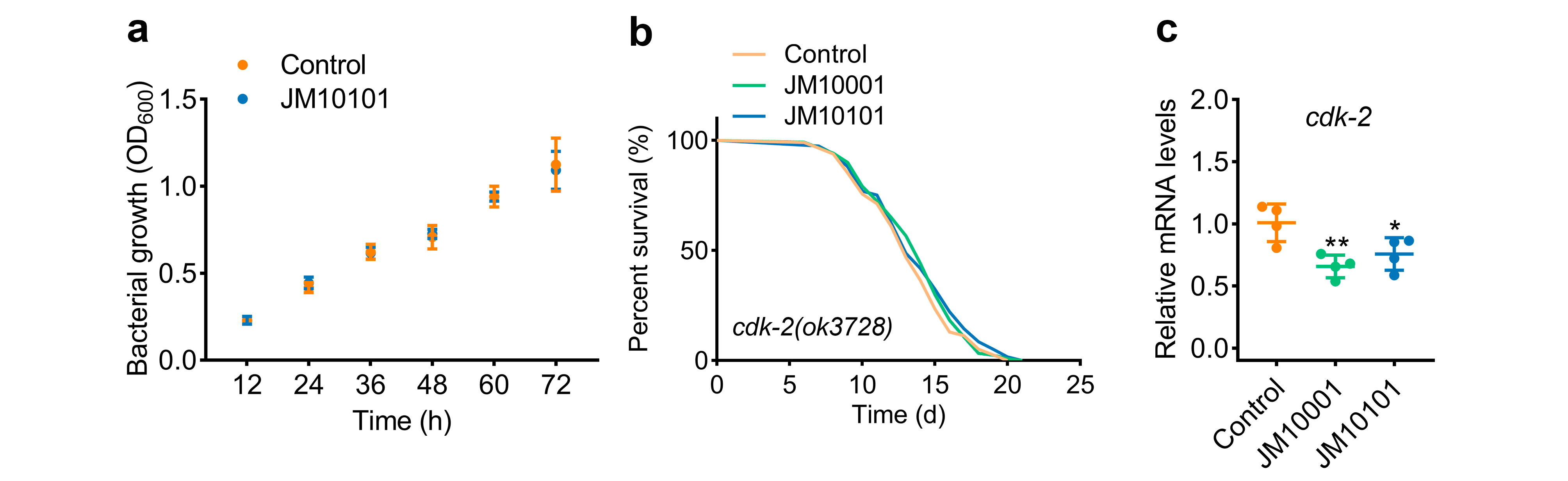


**Figure S5.** (a) Effect of JM10101 in *E. coli* OP50 (*n* = 3). (b) The lifespan of *cdk-2* mutant worms (Control: *n* = 115; JM10001: *n* = 120; JM10101: *n* = 117). (c) The mRNA level of *cdk-2* in WT *C. elegans* (*n* = 4). The concentrations of JM10101 and JM10001 were 0.85 mg/L and 200 mg/L in (a-c), respectively. The data were expressed as the mean ± standard deviation. (a, c) *P* values were calculated using two-sided Student’s t-test; (b) *P* values were calculated using log-rank (Mantel-Cox) test (**P* < 0.05 and ***P* < 0.01).


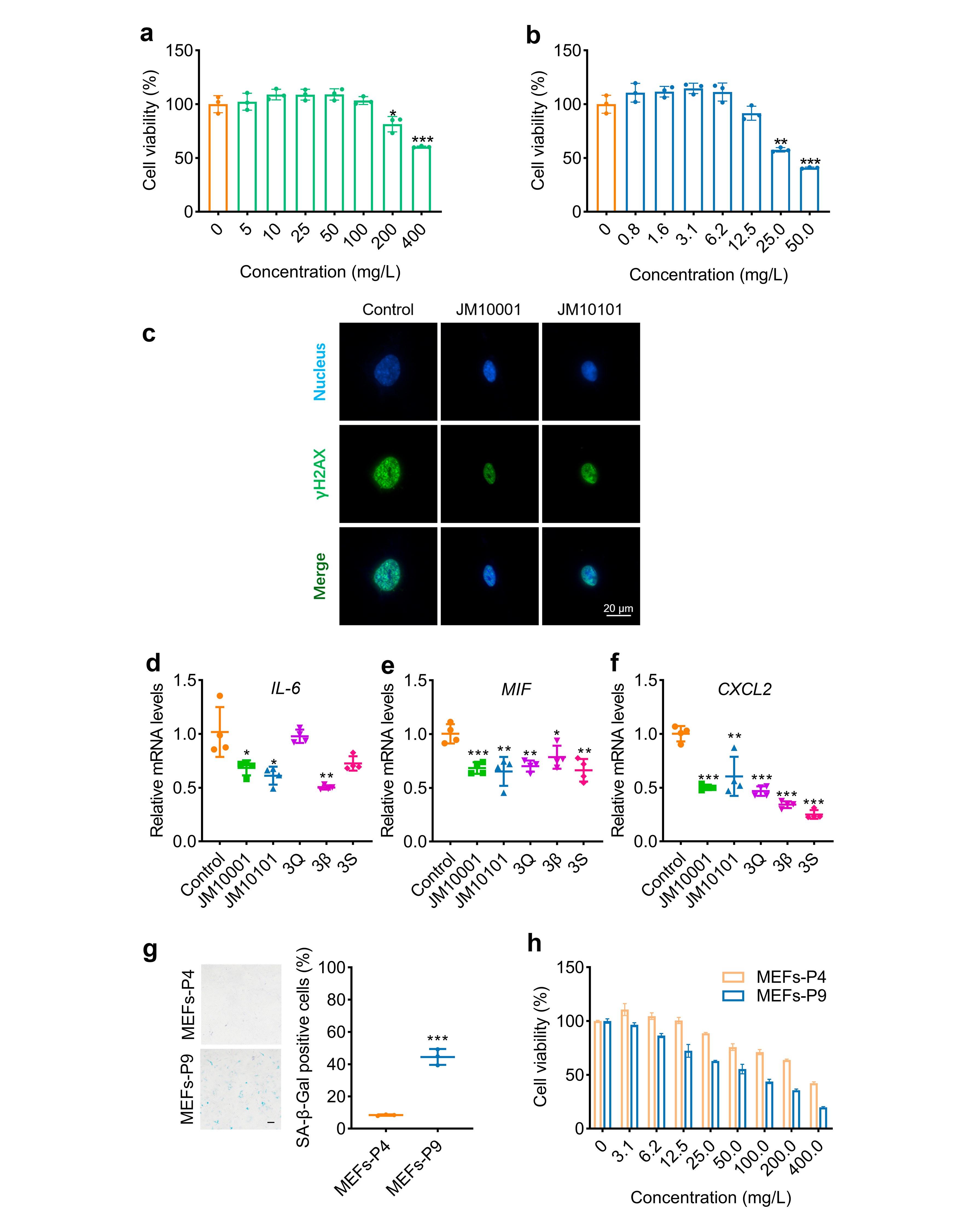


**Figure S6.** Viability of MRC-5 cells in JM10001 (a) and JM10101 (b) at different concentrations (*n* = 3). (c) Representative images of γH2AX staining in MRC-5 cells (P41). (d-f) Reverse transcription quantitative polymerase chain reaction (RT-qPCR) of analysis of SASP factors gene (*IL-6*, *MIF*, *CXCL2*) expression in MRC-5 cells (P39), GAPDH was used as the loading control (*n* = 4). (g) X-gal staining of MEFs cells and quantification of SA-β-gal-positive cells at passage 4 (P4) and passage 4 (P9) (*n* = 3). Scale bar: 100 μm. (h) Cytotoxicity assay of JM10101 to MEFs cells (P4) and MEFs cells (P9) (*n* = 3). The data were expressed as the mean ± standard deviation. (a-b, d-f, h) *P* values were calculated using two-sided Student’s t-test (**P* < 0.05, ***P* < 0.01, and ****P* < 0.001).


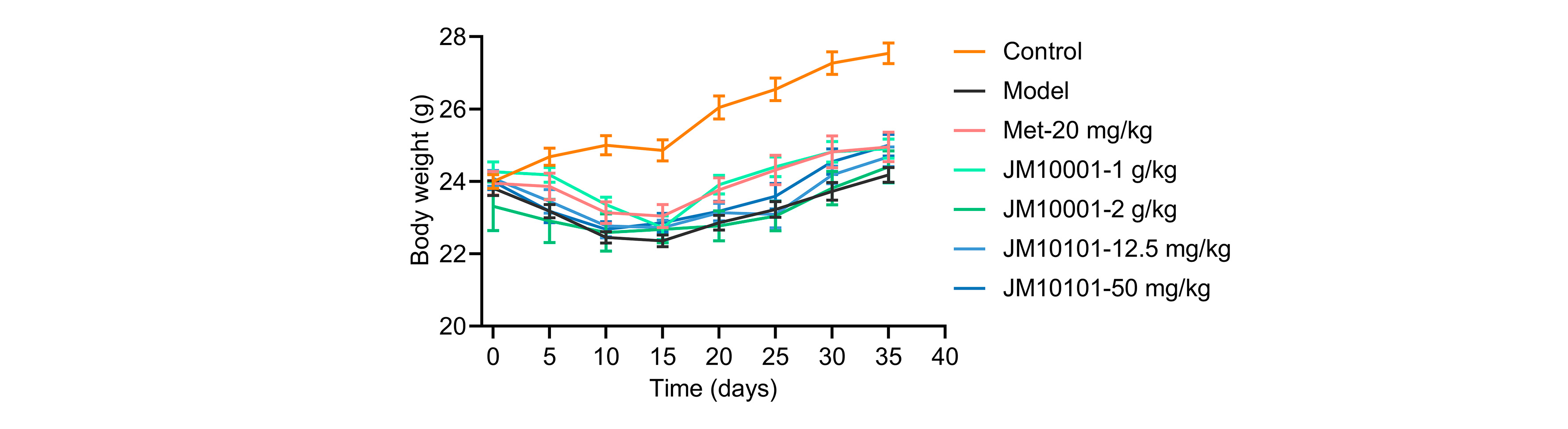


**Figure S7.** Body weight of mice with doxorubicin-induced senescence from each group during the whole observation period. (*n* = 11).


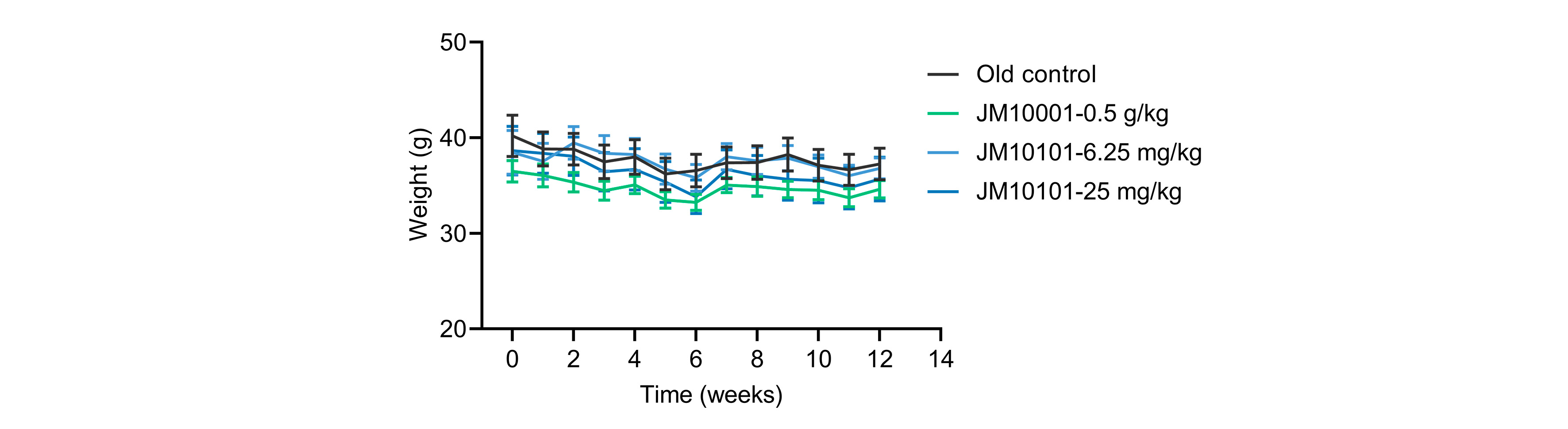


**Figure S8.** Body weight of naturally aged mice model from each group during the whole observation period. (Young control: *n* = 11; Old control: *n* = 9; 0.5 g/kg JM10001: *n* = 10; 6.25 mg/kg JM10101: *n* = 10; 25 mg/kg JM10101: *n* = 10)


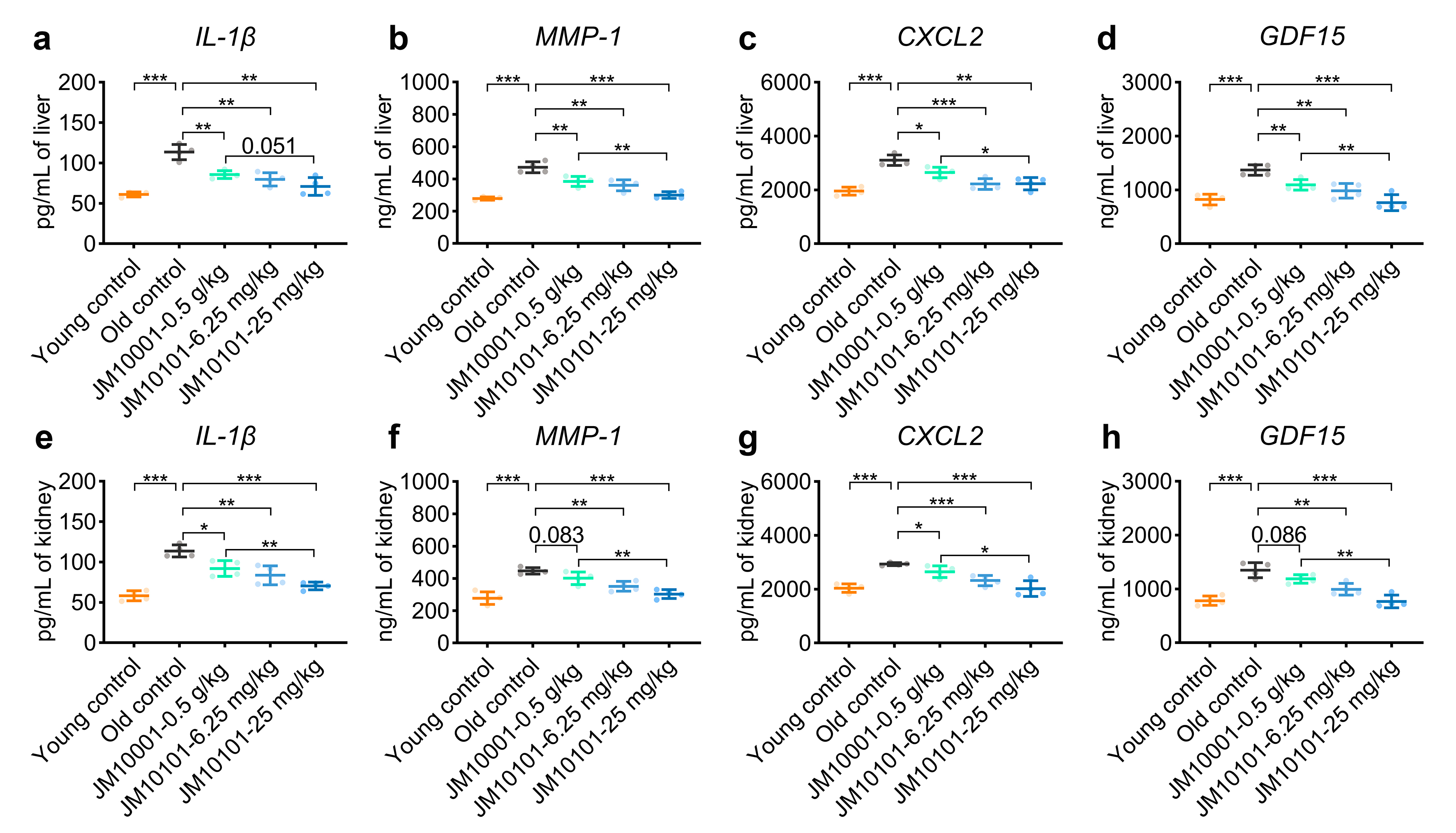


**Figure S9.** The expression of IL-1β (a), MMP-1 (b), CXCL2 (c), and GDF-15 (d) in liver of mice measured by ELISA. The expression of IL-1β (e), MMP-1 (f), CXCL2 (g), and GDF-15 (h) in kidney of mice measured by ELISA (*n* = 4). The data were expressed as the mean ± standard deviation. (a-h) *P* values were calculated using two-sided Student’s t-test (**P* < 0.05, ***P* < 0.01, and ****P* < 0.001).


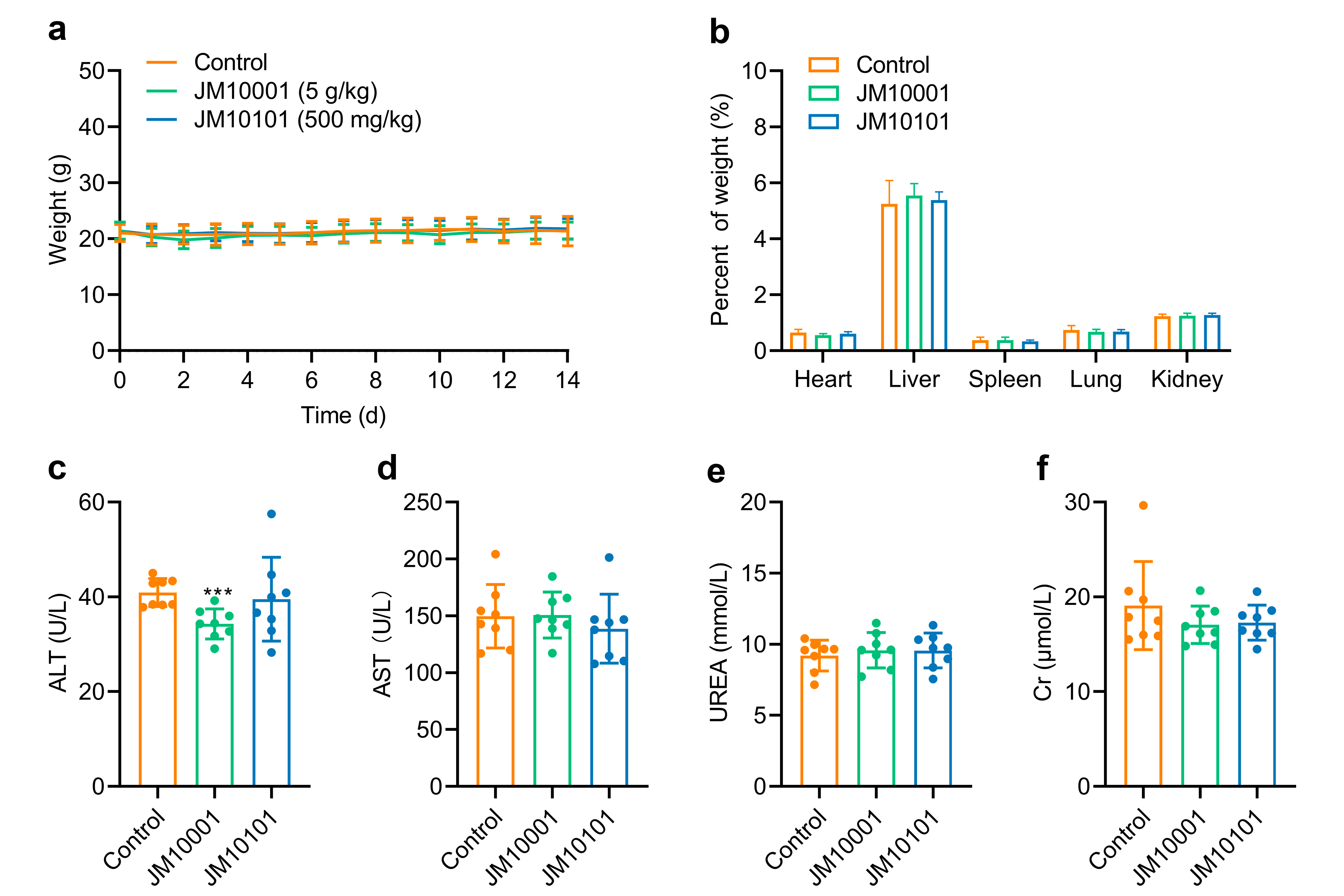


**Figure S10.** Repeat-dose toxicity of JM10001 and JM10101 in mice. (a) The change in weights of mice over a 14-day period. (b) The weight percentage of major organs (heart, liver, spleen, lung, and kidneys) after 14 days of treatment (*n* = 8). (c-f) Liver and renal function of mice were evaluated by serum biochemical parameters (*n* = 8). The concentrations of JM10101 and JM10001 were 500 mg/kg and 5 g/kg in (a-f), respectively (*n* = 8). The data were expressed as the mean ± standard deviation. (a-f) *P* values were calculated using two-sided Student’s t-test (****P* < 0.001).


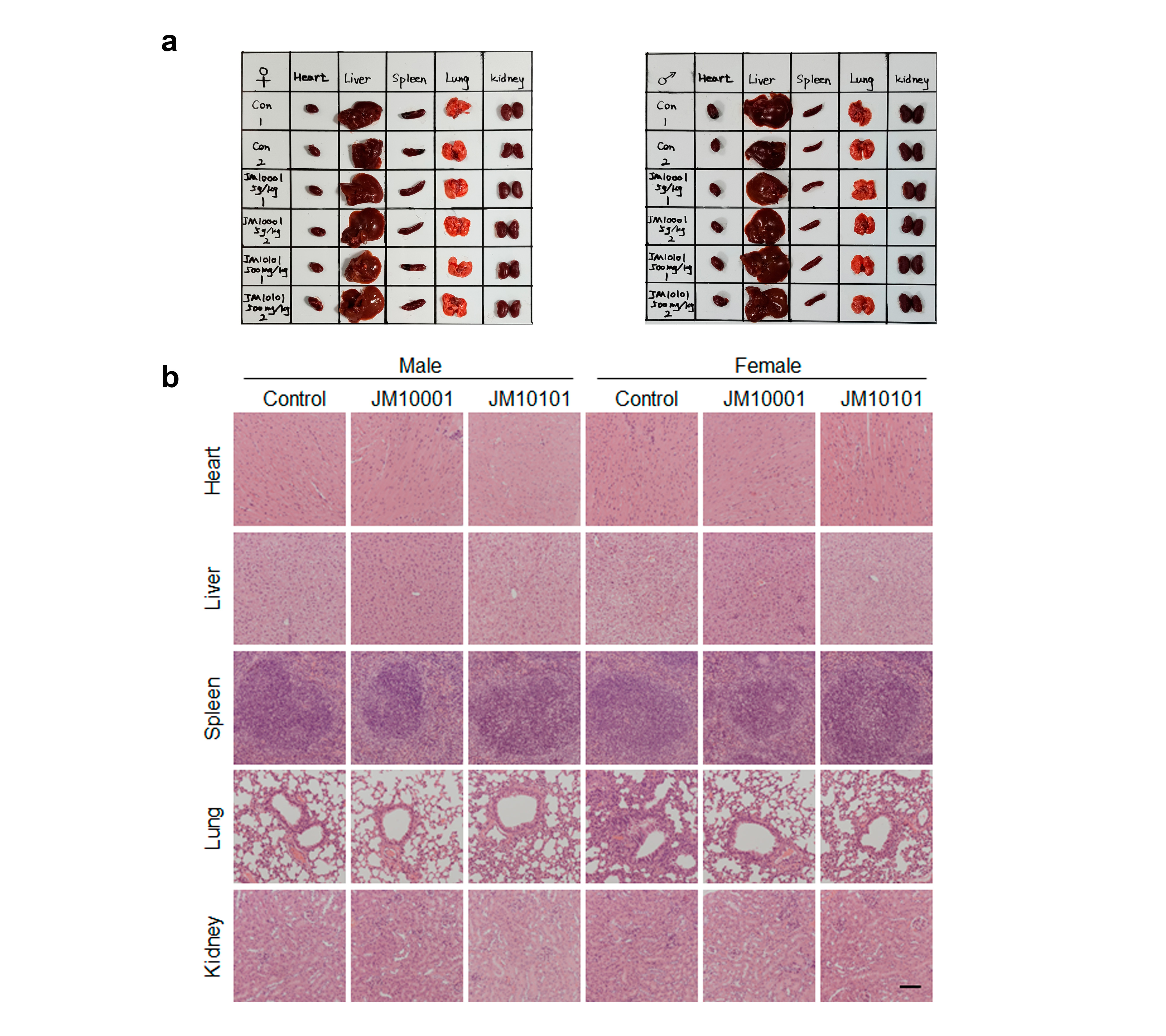


**Figure S11.** (a) Organ morphology of each mouse. (b) Representative pictures of HE staining for major organs in male and female mice. Scale bar: 100 μm. The concentrations of JM10101 and JM10001 were 500 mg/kg and 5 g/kg in (a-b), respectively.

**Supplementary Tables**

**Table S1.** Lifespan data.

| **Figure** | **Strain** | **Genotype** | **Drug**  **treatment** | **Mean lifespan (d)** | **Number of worms** | ***P*-Values** |
| --- | --- | --- | --- | --- | --- | --- |
| Figure 1b | N2 | *WT* | - | 14.53 | 90 | - |
|  |  |  | JM10001  (100 mg/L) | 15.98 | 88 | <0.0001 |
|  |  |  | JM10001  (200 mg/L) | 17.25 | 87 | <0.0001 |
|  |  |  | JM10001  (400 mg/L) | 15.78 | 87 | <0.0001 |
| Figure 2c | TJ1 | *cep-1(gk138)* | - | 16.99 | 79 | - |
|  |  |  | JM10001 (200 mg/L) | 15.90 | 88 | 0.0629 |
| Figure S2d | VC3074 | *cdk-2(ok3728)* | - | 13.40 | 156 | - |
|  |  |  | JM10001 (200 mg/L) | 13.59 | 158 | 0.3474 |
| Figure 3c | N2 | *WT* | - | 11.68 | 120 | - |
|  |  |  | JM10001  (200 mg/L) | 13.22 | 120 | <0.0001 |
|  |  |  | JM10101  (0.85 mg/L) | 13.69 | 118 | <0.0001 |
|  |  |  | Quercetin  (0.30 mg/L) | 12.71 | 119 | <0.001 |
|  |  |  | β-Sitosterol (0.41 mg/L) | 12.79 | 120 | <0.001 |
|  |  |  | Salicylic acid  (0.14 mg/L) | 12.68 | 120 | <0.001 |
| Figure 3h | TJ1 | *cep-1(gk138)* | - | 14.21 | 159 | - |
|  |  |  | JM10001 (200 mg/L) | 13.31 | 159 | <0.05 |
|  |  |  | JM10101  (0.85 mg/L) | 13.87 | 159 | 0.2108 |
| Figure S5b | VC3074 | *cdk-2(ok3728)* | - | 13.22 | 115 | - |
|  |  |  | JM10001 (200 mg/L) | 13.63 | 120 | 0.3576 |
|  |  |  | JM10101  (0.85 mg/L) | 13.69 | 117 | 0.1707 |

***Table S2.*** *UHPLC-Q-Orbitrap MS/MS analysis of* ***JM10001****.*

| **Compound** | **Retention time (min)** | **Adducts** | **Calculate (m/z)** | **Found (m/z)** |
| --- | --- | --- | --- | --- |
| Quercetin | 12.22 | [M-H]^-^ | 301.0354 | 301.0354 |
| β-Sitosterol | 22.48 | [M+Na]^+^ | 437.3754 | 437.3741 |
| Salicylic acid | 8.99 | [M-H]^-^ | 137.0244 | 137.0233 |

**Table S3.** Lifespan data of primary screening (corresponding to Figure 3b).

| **Drug**  **treatment** | **Q : β : S**  **mole ratio** | **Mean lifespan (d)** | **Number of worms** | ***P*-Values** |
| --- | --- | --- | --- | --- |
| - | - | 12.92 | 39 | - |
| JM10101  (0.855 mg/L) | 1:1:1 | 14.69 | 39 | 0.138 |
| JM10102  (1.269 mg/L) | 1:1:4 | 14.24 | 37 | 0.145 |
| JM10103  (2.927 mg/L) | 1:1:16 | 14.51 | 39 | 0.131 |
| JM10104  (9.557 mg/L) | 1:1:64 | 14.54 | 39 | 0.127 |
| JM10105  (1.762 mg/L) | 4:1:1 | 13.89 | 38 | 0.276 |
| JM10106  (2.176 mg/L) | 4:1:4 | 14.93 | 40 | 0.056 |
| JM10107  (3.834 mg/L) | 4:1:16 | 13.90 | 40 | 0.472 |
| JM10108  (10.463 mg/L) | 4:1:64 | 13.79 | 39 | 0.228 |
| JM10109  (5.389 mg/L) | 16:1:1 | 14.20 | 40 | 0.267 |
| JM10110  (5.803 mg/L) | 16:1:4 | 14.60 | 40 | 0.107 |
| JM10111  (7.460 mg/L) | 16:1:16 | 14.36 | 39 | 0.133 |
| JM10112  (14.090 mg/L) | 16:1:64 | 14.03 | 40 | 0.202 |
| JM10113  (19.896 mg/L) | 64:1:1 | 14.20 | 40 | 0.231 |
| JM10114  (20.310 mg/L) | 64:1:4 | 15.39 | 38 | 0.008 |
| JM10115  (21.968 mg/L) | 64:1:16 | 13.95 | 40 | 0.423 |
| JM10116  (28.598 mg/L) | 64:1:64 | 13.70 | 40 | 0.499 |
| JM10117  (2.099 mg/L) | 1:4:1 | 14.46 | 39 | 0.068 |
| JM10118  (2.514 mg/L) | 1:4:4 | 14.40 | 40 | 0.161 |
| JM10119  (4.171 mg/L) | 1:4:16 | 13.82 | 39 | 0.240 |
| JM10120  (10.801 mg/L) | 1:4:64 | 14.20 | 40 | 0.270 |
| JM10121  (3.006 mg/L) | 4:4:1 | 13.55 | 40 | 0.603 |
| JM10122  (3.420 mg/L) | 4:4:4 | 14.44 | 39 | 0.250 |
| JM10123  (5.078 mg/L) | 4:4:16 | 14.39 | 38 | 0.183 |
| JM10124  (11.708 mg/L) | 4:4:64 | 14.68 | 37 | 0.129 |
| JM10125  (6.633 mg/L) | 16:4:1 | 14.40 | 40 | 0.237 |
| JM10126  (7.047 mg/L) | 16:4:4 | 13.33 | 39 | 0.805 |
| JM10127  (8.705 mg/L) | 16:4:16 | 14.18 | 39 | 0.282 |
| JM10128  (15.334 mg/L) | 16:4:64 | 13.95 | 38 | 0.227 |
| JM10129  (21.140 mg/L) | 64:4:1 | 13.48 | 40 | 0.585 |
| JM10130  (21.554 mg/L) | 64:4:4 | 14.95 | 39 | 0.065 |
| JM10131  (23.212 mg/L) | 64:4:16 | 13.86 | 37 | 0.494 |
| JM10132  (29.842 mg/L) | 64:4:64 | 13.78 | 40 | 0.800 |
| JM10133  (7.076 mg/L) | 1:16:1 | 13.98 | 40 | 0.662 |
| JM10134  (7.490 mg/L) | 1:16:4 | 13.32 | 38 | 0.914 |
| JM10135  (9.147 mg/L) | 1:16:16 | 13.51 | 39 | 0.747 |
| JM10136  (15.777 mg/L) | 1:16:64 | 14.05 | 39 | 0.347 |
| JM10137  (7.982 mg/L) | 4:16:1 | 14.61 | 38 | 0.126 |
| JM10138  (8.397 mg/L) | 4:16:4 | 13.60 | 40 | 0.596 |
| JM10139  (10.054 mg/L) | 4:16:16 | 13.63 | 40 | 0.444 |
| JM10140  (16.684 mg/L) | 4:16:64 | 13.88 | 40 | 0.485 |
| JM10141  (11.609 mg/L) | 16:16:1 | 13.58 | 38 | 0.490 |
| JM10142  (12.024 mg/L) | 16:16:4 | 13.69 | 39 | 0.732 |
| JM10143  (13.681 mg/L) | 16:16:16 | 12.78 | 37 | 0.635 |
| JM10144  (20.311 mg/L) | 16:16:64 | 14.08 | 39 | 0.341 |
| JM10145  (26.117 mg/L) | 64:16:1 | 13.97 | 39 | 0.547 |
| JM10146  (26.531 mg/L) | 64:16:4 | 13.05 | 39 | 0.582 |
| JM10147  (28.188 mg/L) | 64:16:16 | 13.82 | 39 | 0.737 |
| JM10148  (34.818 mg/L) | 64:16:64 | 13.13 | 38 | 0.724 |
| JM10149  (26.982 mg/L) | 1:64:1 | 13.40 | 40 | 0.440 |
| JM10150  (27.396 mg/L) | 1:64:4 | 14.58 | 40 | 0.190 |
| JM10151  (29.053 mg/L) | 1:64:16 | 14.46 | 39 | 0.146 |
| JM10152  (35.683 mg/L) | 1:64:64 | 13.26 | 39 | 0.958 |
| JM10153  (27.888 mg/L) | 4:64:1 | 12.08 | 38 | 0.126 |
| JM10154  (28.303 mg/L) | 4:64:4 | 13.78 | 40 | 0.427 |
| JM10155  (29.960 mg/L) | 4:64:16 | 12.80 | 40 | 0.673 |
| JM10156  (36.590 mg/L) | 4:64:64 | 13.28 | 39 | 0.922 |
| JM10157  (31.515 mg/L) | 16:64:1 | 13.10 | 40 | 0.975 |
| JM10158  (31.930 mg/L) | 16:64:4 | 13.46 | 37 | 0.893 |
| JM10159  (33.587 mg/L) | 16:64:16 | 14.44 | 39 | 0.193 |
| JM10160  (40.217 mg/L) | 16:64:64 | 14.10 | 39 | 0.404 |
| JM10161  (46.022 mg/L) | 64:64:1 | 13.47 | 38 | 0.782 |
| JM10162  (46.437 mg/L) | 64:64:4 | 13.13 | 40 | 0.944 |
| JM10163  (48.094 mg/L) | 64:64:16 | 13.45 | 40 | 0.766 |
| JM10164  (54.724 mg/L) | 64:64:64 | 13.46 | 39 | 0.816 |

**Table S4.** Lifespan data of secondary screening (corresponding to Figure 3b).

| **Drug**  **treatment** | **Q : β : S**  **mole ratio** | **Mean lifespan(d)** | **Number of worms** | ***P*-Values** |
| --- | --- | --- | --- | --- |
| **-** | - | 13.89 | 88 | - |
| JM10001  (200 mg/L) | - | 15.11 | 84 | <0.01 |
| JM10101  (0.855 mg/L) | 1:1:1 | 15.39 | 84 | <0.001 |
| JM10102  (1.269 mg/L) | 1:1:4 | 14.39 | 88 | 0.124 |
| JM10103  (2.927 mg/L) | 1:1:16 | 14.23 | 90 | 0.214 |
| JM10104  (9.557 mg/L) | 1:1:64 | 13.80 | 87 | 0.659 |
| JM10106  (2.176 mg/L) | 4:1:4 | 14.51 | 79 | <0.05 |
| JM10110  (5.803 mg/L) | 16:1:4 | 14.54 | 78 | <0.05 |
| JM10111  (7.460 mg/L) | 16:1:16 | 15.21 | 87 | <0.001 |
| JM10114  (20.310 mg/L) | 64:1:4 | 15.01 | 82 | <0.01 |
| JM10117  (2.099 mg/L) | 1:4:1 | 15.12 | 86 | <0.001 |
| JM10118  (2.514 mg/L) | 1:4:4 | 15.01 | 83 | <0.01 |
| JM10122  (3.420 mg/L) | 4:4:4 | 14.15 | 87 | 0.163 |
| JM10123  (5.078 mg/L) | 4:4:16 | 14.57 | 88 | <0.05 |
| JM10124  (11.708 mg/L) | 4:4:64 | 14.42 | 86 | 0.061 |
| JM10125  (6.633 mg/L) | 16:4:1 | 15.28 | 81 | <0.0001 |
| JM10130  (21.554 mg/L) | 64:4:4 | 15.19 | 88 | <0.001 |
| JM10137  (7.982 mg/L) | 4:16:1 | 14.51 | 80 | <0.05 |
| JM10150  (27.396 mg/L) | 1:64:4 | 14.56 | 84 | <0.05 |
| JM10151  (29.053 mg/L) | 1:64:16 | 14.90 | 82 | <0.001 |
| JM10159  (33.587 mg/L) | 16:64:16 | 14.58 | 84 | <0.001 |

**Table S5.** Primers for specific genes.

| **Genes** | **Species** | **Forward Primer (5’ to 3’)** | **Reverse Primer (5’ to 3’)** |
| --- | --- | --- | --- |
| *GADPH* | *C. elegans* | GGAACTGTTACCTACGATGGAGA | AAACTCCAGTAGACTCGACAACG |
| *sod-1* |  | CGTAGGCGATCTAGGAAATGTG | TGACGAGCGTGTCGGTGAG |
| *sod-2* |  | GATACTGTCCAAAGGGAAAGAT | GTAGTAAGCGTGCTCCCAGA |
| *sod-3* |  | ATCTACTGCTCGCACTGCTT | TTTCATGGCTGATTACAGGTT |
| *sod-4* |  | GCACCAGATGACTCGAACA | GTCCACTTAATGAGGCAAGA |
| *sod-5* |  | TCGAAACGTGCTGTAGCGG | CACCTTCGGCTTTCTGGGT |
| *cdk-2* |  | GCCTTCGTGAAATTTCGTGTAT | AATGACTTCACATAGTTTGGCG |
| *GADPH* | *Homo sapiens* | GGAACTGTTACCTACGATGGAGA | AAACTCCAGTAGACTCGACAACG |
| *p53* |  | GACAAGATGGAGAGAGTGGATC | TTGAAGCGCTTAATATCCTGGA |
| *p21* |  | GATCGAATTAGCTCGCGTAATC | TCCGCTAATGTATTACGTTCCA |
| *IL-1β* |  | ATGATGGCTTATTACAGTGGCAA | GTCGGAGATTCGTAGCTGGA |
| *MMP-1* |  | AGCCTTCCAACTCTGGAGTAATGT | CCGATGATCTCCCCTGACAA |
| *MMP-2* |  | ATGCCGTCGTGGACCTGC | TGCTTCCAAACTTCACGCTCTT |
| *CCL-2* |  | AAGACCATTGTGGCCAAGGA | TTCGGAGTTTGGGTTTGCT |
| *IL-6* |  | ACTCACCTCTTCAGAACGAATTG | CCATCTTTGGAAGGTTCAGGTTG |
| *MIF* |  | ATCGTAAACACCAACGTGCC | TTGCTGTAGGAGCGGTTCTG |
| *CXCL-2* |  | AGAATGGGCAGAAAGCTTGTCT | CCTTCTGGTCAGTTGGATTTGC |
