# Supplementary material for: Correction to “A Senomorphlytic Three‐Drug Combination Discovered in Salsola collina for Delaying Aging Phenotypes and Extending Healthspan”
Source: Adv Sci (Weinh). 2025 Apr 25;12(19):2505321. doi: 10.1002/advs.202505321 (PMC12097131; doi:10.1002/advs.202505321)
Supplement: Supplementary file 1 — Supporting Information [file ADVS-12-2505321-s001.docx]

Supporting Information

for Adv. Sci., DOI 10.1002/advs.202401862

**Correction to “A Senomorphlytic Three-Drug Combination Discovered in *Salsola collina* for Delaying Aging Phenotypes and Extending Healthspan”**

*Jiqun Wang, Wenwen Liu, Yunyuan Huang, Guangwei Wang, Xiaobo Guo, Donglei Shi, Tianyue Sun, Chaojiang Xiao, Chao Zhang, Bei Jiang, Yuan Guo, and Jian Li.*

Raw data of Figure 6b:

| ALT (U/L) | | | | | | |
| --- | --- | --- | --- | --- | --- | --- |
| Control | Model | 20 mg/kg  Met | 1 g/kg  JM10001 | 2 g/kg  JM10001 | 12.5 mg/kg  JM10101 | 50 mg/kg  JM10101 |
| 32 | 30 | 36 | 51 | 32 | 30 | 44 |
| 30 | 54 | 33 | 33 | 32 | 28 | 45 |
| 30 | 59 | 29 | 34 | 27 | 49 | 28 |
| 28 | 45 | 33 | 28 | 28 | 27 | 24 |
| 29 | 41 | 37 | 30 | 25 | 39 | 39 |
| 41 | 33 | 36 | 31 | 33 | 43 | 29 |
| 34 | 30 | 35 | 32 | 50 | 27 | 39 |
| 40 | 68 | 26 | 35 | 34 | 36 | 36 |
| 32 | 35 | 45 | 49 | 27 | 30 | 25 |
| 41 | 73 | 32 | 32 | 37 | 64 | 40 |
| 32 | 41 | 38 | 34 | 30 | 36 | 31 |
